# Anti-rheumatic colchicine phytochemical exhibits potent antiviral activities against avian and seasonal Influenza A viruses (IAVs) via targeting different stages of IAV replication cycle

**DOI:** 10.1186/s12906-023-04303-2

**Published:** 2024-01-22

**Authors:** Akram Hegazy, Raya Soltane, Ahlam Alasiri, Islam Mostafa, Ahmed M. Metwaly, Ibrahim H. Eissa, Sara H. Mahmoud, Abdou Kamal Allayeh, Noura M. Abo Shama, Ahmed A. Khalil, Ramya S. Barre, Assem Mohamed El-Shazly, Mohamed A. Ali, Luis Martinez-Sobrido, Ahmed Mostafa

**Affiliations:** 1 Department of Agricultural Microbiology, Faculty of Agriculture, Cairo University, Giza, 12613 Giza District Egypt; 2 Department of Biology, Adham University College, Umm Al-Qura University, 21955 Makkah, Saudi Arabia; 3 Department of Pharmacognosy, Faculty of Pharmacy, Zagazig University, Zagazig, 44519 Egypt; 4 Pharmacognosy and Medicinal Plants Department, Faculty of Pharmacy (Boys), Al-Azhar University, Cairo, Egypt; 5 Biopharmaceutical Products Research Department, Genetic Engineering and Biotechnology Research Institute, City of Scientific Research and Technological Applications (SRTA-City), Alexandria, 21934 Egypt; 6 Pharmaceutical Medicinal Chemistry & Drug Design Department, Faculty of Pharmacy (Boys), Al-Azhar University, Cairo, 11884 Egypt; 7 Center of Scientific Excellence for Influenza Viruses, National Research Centre, Giza, 12622 Egypt; 8 Virology Lab 176, Water Pollution Research Department, Environment and Climate Change Institute, National Research Centre, Dokki, 12622 Giza Egypt; 9 Agriculture Research Center (ARC), Veterinary Sera and Vaccines Research Institute (VSVRI), Cairo, 11435 Egypt; 10 Texas Biomedical Research Institute, San Antonio, TX USA; 11 Faculty of Pharmacy, El Saleheya El Gadida University, El Saleheya El Gadida , Sharkia, 44813 Egypt

**Keywords:** Antivirals, Alkaloids, Colchicine, Avian Influenza Virus (AIV), Seasonal Influenza

## Abstract

**Background:**

The continuous evolution of drug-resistant influenza viruses highlights the necessity for repurposing naturally-derived and safe phytochemicals with anti-influenza activity as novel broad-spectrum anti-influenza medications.

**Methods:**

In this study, nitrogenous alkaloids were tested for their viral inhibitory activity against influenza A/H1N1 and A/H5N1 viruses. The cytotoxicity of tested alkaloids on MDCK showed a high safety range (CC_50_ > 200 µg/ml), permitting the screening for their anti-influenza potential.

**Results:**

Herein, atropine sulphate, pilocarpine hydrochloride and colchicine displayed anti-H5N1 activities with IC_50_ values of 2.300, 0.210 and 0.111 µg/ml, respectively. Validation of the IC_50_ values was further depicted by testing the three highly effective alkaloids, based on their potent IC_50_ values against seasonal influenza A/H1N1 virus, showing comparable IC_50_ values of 0.204, 0.637 and 0.326 µg/ml, respectively. Further investigation suggests that colchicine could suppress viral infection by primarily interfering with IAV replication and inhibiting viral adsorption, while atropine sulphate and pilocarpine hydrochloride could directly affect the virus in a cell-free virucidal effect. Interestingly, the in silico molecular docking studies suggest the abilities of atropine, pilocarpine, and colchicine to bind correctly inside the active sites of the neuraminidases of both influenza A/H1N1 and A/H5N1 viruses. The three alkaloids exhibited good binding energies as well as excellent binding modes that were similar to the co-crystallized ligands. On the other hand, consistent with in vitro results, only colchicine could bind correctly against the M2-proton channel of influenza A viruses (IAVs). This might explicate the in vitro antiviral activity of colchicine at the replication stage of the virus replication cycle.

**Conclusion:**

This study highlighted the anti-influenza efficacy of biologically active alkaloids including colchicine. Therefore, these alkaloids should be further characterized in vivo (preclinical and clinical studies) to be developed as anti-IAV agents.

## Introduction

Influenza is a highly contagious respiratory tract infection that threatens the lives of many people all over the world [1]. Influenza viruses (IVs) possess a segmented RNA genome of negative sense [2]. They belong to the Orthomyxoviridae family and divide into four types: influenza A viruses (IAVs), influenza B viruses (IBVs), influenza C viruses (ICVs) and influenza D viruses (IDVs) [3]. Due to high genetic dynamism, the continuous tendency to antigenic drift/shift, efficient viral transmission, the rapid emergence of drug resistance and limited efficacy of currently available medications, the members of IAV genus are considered major life-threatening respiratory pathogens as it is responsible for all documented influenza pandemics [4, 5]. Remarkably, the estimated number of deaths caused by the influenza A/H1N1 virus during the “Spanish influenza” pandemic from 1918 to 1920 (> 50 million deaths) exceeded those caused by coronavirus disease 2019 (COVID-19) (6.9 million deaths) [6, 7]. Another member of the IAV genus, the avian influenza A/H5N1virus, is characterized by an extremely high mortality rate in poultry and human populations [8]; hence it is considered a highly pathogenic avian influenza virus (HPAIV) [9, 10].

Vaccination and antiviral therapy are the main strategies used worldwide to control influenza infection in humans. However, the effectiveness of available vaccines to control seasonal influenza infection is relatively low due to different factors such as the suitability of the vaccine to the viral strain, transmission across species and sudden genetic mutations to the vaccine strain [11–13]. Moreover, the antiviral resistance and the emergence of mutant viral strains have reduced the efficacy of the known FDA-approved synthetic antiviral agents such as M2-channel blockers and neuraminidase inhibitors (NAIs) [14–16]. Therefore, antiviral research should be prioritized to discover new alternatives for the prophylaxis and control of IAV infections. Phytochemicals derived from plants and natural resources are considered one of the safest and most efficacious treatment options for controlling viral infections including influenza [17–21]. The largest known class of phytochemicals are called alkaloids which were documented for the first time, over 4 thousand years ago [22]. These basic compounds consist of one or more nitrogen atoms bound to a heterocyclic nucleus [23]. Higher plants especially those belonging to Ranunculaceae, Leguminosae, Papaveraceae, Menispermaceae, and Loganiaceae families are considered main reservoirs for alkaloids [24]. Alkaloids can serve as therapeutic options for the treatment of a variety of diseases and exhibit promising biological activities [25, 26].

Pharmacoinformatics have been forefront of the drug design and development research. They have decreased the expense, time, and labor of drug discovery [27]. Hence, computational chemistry methods were applied to estimate various pharmacodynamic and pharmacokinetic parameters that relate the chemical structure of compounds to their activity and to characterize the interaction of compounds with biological targets [28–30].

Herein, the anti-influenza activities of some alkaloid compounds were investigated against two different subtypes of IAVs with varied host ranges: the avian influenza A/H5N1 and the seasonal influenza A/H1N1 viruses to help in stockpiling of antiviral medications to be ready for any future pandemic situation. In addition, a molecular docking approach was applied to investigate the binding patterns of the active compounds against the prospective biological targets (influenza H1N1 neuraminidase, influenza H5N1 neuraminidase, and influenza M2).

## Materials and methods

### Cell lines and viruses

The MDCK (Madin Darby Canine Kidney) cell line, provided by the Center of Scientific Excellence for Influenza Viruses (CSEIV), National Research Centre (NRC), Egypt, were cultured at 37°C and 5% CO_2_ under humified conditions using the growth medium (GM). The GM is composed of Dulbecco’s Modified Eagle’s Medium (DMEM) (DMEM; BioWhittaker, Walkersville, MD, USA) including 5% fetal bovine serum (FBS) (Gibco-BRL; New York, USA) and 1% Penicillin/Streptomycin (pen/strep) antibiotic/antimycotic mixture (GIBCO-BRL; New York, USA). In the same line, the seasonal influenza A/Egypt/NRC098/2019(H1N1) (GISAID ID: EPI_ISL_12995118) and highly pathogenic avian influenza A/chicken/Egypt/N12640A/2016 (H5N1) obtained from the virus collections of the CSEIV, NRC, Egypt, were propagated in confluent MDCK cell monolayers [31, 32] and titrated using plaque infectivity assay (PIA) and Tissue Culture Infectious Dose (TCID_50_) method [33–35].

### Viral titration

#### Median Tissue Culture Infectious Dose (TCID_50_) Method

To determine the viral dilutions that can infect 50% of the MDCK cell line, we conducted the median tissue culture infectious dose (TCID_50_) according to Reed and Muench method [33, 35]. Briefly, serial decimal viral dilutions, in triplicates, were applied into MDCK cell monolayers in 96 well plates and incubated at 37°C in a humidified 5% CO_2_ incubator. At 72 h post incubation, infected and control cell monolayers were fixed using a 10% paraformaldehyde (10% PFA) solution. Following fixation and drying the cell monolayers, a volume of 100 µL crystal violet stain (0.1% in 25% methanol, abbreviated hereafter as CV) was added to each well and allowed to incubate at room temperature (RT) for 20 min. Plates were next thoroughly washed with water to remove excess staining material and dried overnight. Once dried, plates were assessed for cytopathic effect (CPE) in each column. The final titer was calculated using the Reed–Muench Method [33].

#### Plaque Infectivity Assay (PIA)

To determine the countable virus titer in plaque forming unit (PFU)/ml, a plaque infectivity assay was used as previously described [34], with minor modifications. Briefly, MDCK cells were subcultured and incubated overnight under the optimal growth conditions, as previously stated, into 6-well cell culture plates. The MDCK monolayers (80–90% confluency) underwent washing with 1X phosphate buffer saline (PBS) and were further loaded with successive decimal dilutions (tenfold) of IAV in viral infection medium (VIM) (1X DMEM supplemented with 4% bovine serum albumin (BSA) (Gibco-BRL; New York, USA), 1% pen/strep mixture and 1 µg/mL of L‐1‐tosyl‐ amido‐2‐phenylethyl chloromethyl ketone (TPCK)-treated trypsin for 1 h at 37 °C in a humified 5% CO_2_ incubator to allow viral adsorption. Meanwhile, the plates were manually agitated every 15 min for even distribution of the inocula. Subsequently, the remains of the inocula were aspirated and the cells were covered with 2 ml overlay medium containing 1% agarose, 1X DMEM, 4% bovine serum albumin (BSA) (Gibco-BRL; New York, USA), 1% pen/strep mixture and 1 µg/mL TPCK-treated trypsin. The plates were allowed to be set, then incubated at 37 °C in a humidified 5% CO_2_ incubator for 60–72 h. Finally, the plates underwent virus inactivation and cell monolayer fixation with 10% PFA and visualization of plaques via CV staining as described above. The calculations of the viral titres were employed using the next formula:

$$\text{PFU}/\mathrm{mL}=\mathrm{Number}\;\mathrm{of}\;\mathrm{plagues}\;\times\;\mathrm{Reciprocal}\;\mathrm{of}\;\mathrm{virus}\;\mathrm{dilution}\;\times\;\mathrm{Dilution}\;\mathrm{factor}\;\left(\mathrm{to}\;\mathrm{reach}\;1\mathrm m\right)$$

### Phytochemicals (Alkaloids)

The investigated alkaloids in the current study are listed in Table 1. Hyoscyamine, trigonelline hydrochloride, scopolamine hydrochloride, atropine sulphate, quinidine sulphate and pilocarpine hydrochloride were purchased from Sigma-Aldrich Chemie GmbH Eschenstr. 5, Taufkirchen Germany. Papaverine hydrochloride was obtained from Recordati industria chimicae farmaceutica s.p.a., Milano, Italy. Quinine sulphate was purchased from Loba Chemie Pvt Ltd, Mumbai, India. Caffeine was obtained from C.H. Boehringer Sohn Ingelheim, Ingelheim am Rhein, Germany. Ephedrine hydrochloride was provided by Alexandria Company for Pharmaceuticals and Chemical Industries, As Soyou Qebli, Alexandria, Egypt. Colchicine was obtained from El Nasr Pharmaceutical Chemicals Co., Abu Zaabal, Egypt.

**Table 1:** The chemical classification and biological activities of the alkaloids and reference anti-influenza drugs used in this study

Compound No	Compound name	CAS No	Class	Biological Activities	Ref
1	1-Hyoscyamine	101–31-5	Tropane Alkaloids	Antiviral	[36]
2	Trigonelline HCl	6138–41-6	Pyridine Alkaloids	Neuroprotective, anti-degranulation, antibacterial, antimicrobial, antiviral, anti-tumour	[37–39]
3	(-)Scopolamine HCl	55–16-3	Tropane Alkaloids	Anticholinergic	[40]
4	Papaverine HCl	61–25-6	Benzyl isoquinoline Alkaloids	Antiviral	[41–43]
5	Atropine sulphate	55–48-1	Tropane Alkaloids	Anticholinergic, anticonvulsant, antiviral	[39, 44]
6	Quinine sulphate	50–54-4	Quinoline Alkaloids	Antiprotozoal, antiviral	[45–47]
7	Caffeine	58–08-2	Xanthine (Purine base) Alkaloids	CNS stimulant, anti-inflammatory, analgesic, anticarcinogenic, antiviral	[48–53]
8	Ephedrine HCl	50–98-6	Amino Alkaloids	Anticarcinogenic, antiviral	[54, 55]
9	Quinidine sulphate	50–54-4	Quinoline Alkaloids	Antidepressant	[56]
10	Pilocarpine HCl	54–71-7	Imidazole Alkaloids	Anticholinergic	[57]
11	Colchicine	64–86-8	Tropolone Alkaloids	Anti-inflammatory, antiviral	[58–60]
12	Zanamivir	139110–80-8	NAI (Drug control)	FDA-approved anti-influenza drug	[61]

### Cytotoxicity and antiviral assay

To assess the toxicity of the tested phytochemicals on MDCK cells and their antiviral inhibitory effects against the tested IAVs, the CC_50_ (half maximal cytotoxic concentration) and IC_50_ (half maximal inhibitory concentration) were performed using crystal violet assay [35, 62]. Briefly, MDCK cells were cultivated in 96-well plates and incubated for 24 h as previously described. The next day, the cultured plates underwent washing with 1X PBS and incubated (under humified conditions at 37°C/5% CO_2_) for 72 h with triplicates of the serial decimal dilutions of the tested phytochemicals in VIM. Three wells with MDCK monolayers were kept untreated without adding any compound to be used as control wells. Three days later, the treated and untreated cell monolayers were subjected to fixation with 10% PFA and visualized with CV solution. The plates were then washed out and dried overnight at RT. To dissolve the CV stain, 100 µl of absolute methanol was dispensed in each well and the plates were then shaken for 20 min. The optical density was then measured at 570 nm wavelength using an Anthos Zenyth 200rt reader (Anthos Labtec Instruments, Heerhugowaard, Netherlands).

To determine the IC_50_ of each compound as described previously [35], MDCK cells were cultured into cell monolayers in 96-wells cell culture plates. The exhausted media were aspirated and the cell monolayers were washed with 1X PBS and incubated with the tested viruses at RT for 1 h to permit viral adsorption into MDCK cells. Subsequently, the non-cytotoxic concentrations of each compound were added to the infected cells (100 μl/well), keeping three infected/untreated wells to be used as virus control and another three uninfected/untreated wells as cell control. The plates were further incubated at 37°C under humified 5% CO_2_ conditions. At 72 h post-infection and treatment, the cell monolayers were fixed with 100 μl of 10% PFA for 1 h and stained with CV solution at RT for 20 min. CV stain in overnight-dried plates was dissolved in 100 μl of absolute methanol to allow OD measurement at 570 nm wavelength via Anthos Zenyth 200rt reader (Anthos Labtec Instruments, Heerhugowaard, Netherlands).

### Plaque Reduction Assay (PRA)

Following IC_50_ determination, a modified plaque reduction assay [35] was employed to assure the antiviral activity of the three potent alkaloids. Briefly, non-cytotoxic concentrations of each potent alkaloid (previously determined through CC_50_ determination) were added to the predetermined viral dilutions (countable dilution) in VIM (enriched with 1 μg/mL TPCK-treated trypsin for the A/H1N1 virus) and incubated at 25°C /1h. Following the incubation, triplicates of the virus/compound mixture were incubated for 1 h with 80–90% confluent MDCK cells at 37°C under humified 5% CO_2_ conditions to permit viral adsorption. The supernatants were then removed and the cells were covered with the overlay medium. The plates were then incubated at 37°C in a humified 5% CO_2_ incubator for 72 h. Finally, the plates were subjected to fixation and visualization as previously described in the plaque infectivity method. The plaque reduction percentages were calculated utilizing the next formula:

$$Viral\;reduction\;(\%)=\frac{\mathrm{Count}\;\mathrm{of}\;\mathrm{untreated}\;\mathrm{viru}s\;(\text{control})-\mathrm{Count}\;\mathrm{of}\;\mathrm{treated}\;\mathrm{virus}}{\mathrm{Count}\;\mathrm{of}\;\mathrm{untreated}\;\mathrm{virus}\;(\text{control})}\times100$$

### Stages of antiviral action

To reveal the stage(s) of antiviral action for each alkaloid with low IC_50_ and high selectivity index (SI) values against the HPAIV (A/H5N1), minor changes have been made to the plaque reduction assay described earlier [35]. The antiviral activity can either be performed via suppressing viral replication or interfering with viral adsorption or directly targeting the viral particles themselves in cell-free status (virucidal action).

#### Interference with viral replication

Typically, 80–90% confluent monolayers of MDCK cells in 6-well plates were infected with 100 μl/well of the countable viral dilution of influenza A/H5N1 virus, taking into consideration the untreated control cell and virus control and incubated at 37°C for 1 h in a humidified 5% CO_2_ incubator. The remaining inocula including non-adsorbed viral particles were removed via washing with 1X PBS solution. Thereafter, the predefined safe concentrations for each highly potent compound were added and another short incubation time was applied under the same conditions for 1 h. The plates were washed once more and finally covered with 2 ml of the overlay medium and incubated at 37°C for 72 h in a humified 5% CO_2_ incubator. Following the incubation, the fixation and visualization steps were conducted as described in the plaque infectivity assay.

#### Interference with viral adsorption

This assay was conducted to permit chemical adsorption onto host cell receptors without internalization. In brief, the MDCK cells (80–90% confluency) previously seeded in 6-well plates were treated with the safe concentrations of each potent alkaloid where cell and virus control wells were included. The plates were then incubated at 4°C for 1h to allow interaction between compounds and host cell receptors without active internalization of the phytochemical. The residual non-adsorbed compounds were then aspirated and the plates underwent washing using 1X PBS. After washing, the cells were infected with the countable viral dilution of influenza A/H5N1 virus and the plates were incubated once more at 37°C for 1h in a humidified 5% CO_2_ incubator. Another washing with 1X PBS was applied to remove the non-adsorbed viral particles. Finally, the cells were covered with 2 ml overlay medium and incubated at 37°C for 72 h under humidified 5% CO_2_ conditions. Following the incubation, the fixation and visualization steps were conducted as described in the plaque infectivity assay.

#### Virucidal action

Non-cytotoxic active concentrations of each potent compound were applied to 3–4 times higher dilution than the countable dilution of the IAV and incubated at 25°C for 1h. Next, a successive 3–4 times serial decimal dilution was employed to reach the countable titer of the influenza A/H5N1 virus. Thereafter, 80–90% confluent monolayers of the MDCK cells in 6-well plates were then treated with the diluted virus/compound mixture (including neglectable phytochemical concentration but countable virus dilution) for 1 h at 37°C under humified 5% CO_2_ conditions. Then, a washing step with 1X PBS was applied and the cells were covered with 2 ml overlay medium and incubated at 37°C for 72 h in a humidified 5% CO_2_ incubator. Finally, fixation and visualization steps were conducted as described in the plaque infectivity assay.

### In silico docking studies

#### Protein preparation

The crystal structures of influenza A/H1N1 neuraminidase (PDB ID: 6HP0, resolution: 1.88 Å), influenza A/H5N1 neuraminidase (PDB ID: 3CKZ, resolution: 1.90 Å), and influenza M2 (PDB ID: 2RLF) were obtained from Protein Data Bank (https://www.rcsb.org). At first, the crystal structures of the IAV N1-type neuraminidases from H1N1 and H5N1 subtypes, and IAV M2 complexed with the co-crystallized ligands (GJT, zanamivir, and rimantadine, respectively) were prepared by removing crystallographic water molecules. Only one chain for influenza A/H1N1-subtype and influenza A/H5N1-subtype N1 neuraminidase was retained besides the co-crystallized ligands. For IAV M2, we used all chains in the docking process. The selected protein chains were protonated using the following setting. The used electrostatic functional form was GB/VI with a distance cut-off of 15 Å. The used value of the dielectric constant was 2 with an 80 dielectric constant of the used solvent. The used Van der Waals functional form was 800R3 with a distance cut-off of 10 Å. Then, the energy of the protein chains was minimized using Hamiltonian AM1 implanted in Molecular Operating Environment (MOE 2019 and MMFF94x (Merck molecular force field) for structural optimization. Next, the active sites of the target proteins were defined for ligand docking and redocking (in case of validation of docking protocol). The active sites of the proteins were identified as the residues that fall within the 5 Å distance from the perimeter of the co-crystallized ligand [63].

#### Ligand preparation

The 2D structures of the tested molecules and the reference compounds were drawn using ChemBioDraw Ultra 14.0 and saved in MDL-SD file format. The tested ligand were used as follows. i) atropine ((1*R*,5*S*)-8-methyl-8-azabicyclo[3.2.1]octan-3-yl 3-hydroxy-2-phenylpropanoate), ii) pilocarpine ((3*S*,4*R*)-3-ethyl-4-((1-methyl-1*H*-imidazol-5-yl)methyl)dihydrofuran-2(3*H*)-one), and iii) colchicine ((*S*)-*N*-(1,2,3,10-tetramethoxy-9-oxo-5,6,7,9-tetrahydrobenzo[a]heptalen-7-yl)acetamide). Since in silico docking of racemic molecules is not feasible, we carried out the docking studies for a single isomer as appeared in the chemical names of the tested ligands [64]. Each tested ligand was specified as a single isomer using MOE builder. Then, the 3D structures of the ligands were protonated and optimized by energy minimization using MM2 force-field and 10,000 iteration steps of 2 fs. The conformationally optimized ligands were used for docking studies [65].

#### Docking setup and validation of docking protocol

The protein–ligand docking studies were carried out using MOE version 2019. Validation of the docking protocol was carried out by redocking the co-crystallized reference ligands (GJT and zanamivir) against the isolated pockets of IAV N1-type neuraminidases from H1N1 and H5N1 IAV subtypes, and IAV M2, respectively. The docking protocol was validated by comparing the RMSD values of the re-docked ligand poses with the corresponding co-crystallized reference ligand structures.

The docking setup for the tested compounds was established according to the protocol followed in the validation step. For each docking run, 30 docked solutions were generated using ASE for scoring function and rigid receptor for refinement. The pose with an ideal binding mode was selected for further investigations. The docking results were visualized using Discovery Studio (DS) 4.0. Analysis of the docking results was carried out by comparing the interactions and docking scores obtained for the docked ligands with that of the re-docked reference molecules [66, 67].

## Results

### Cytotoxicity and viral inhibitory effects of the tested alkaloids

**Fig. 1:**
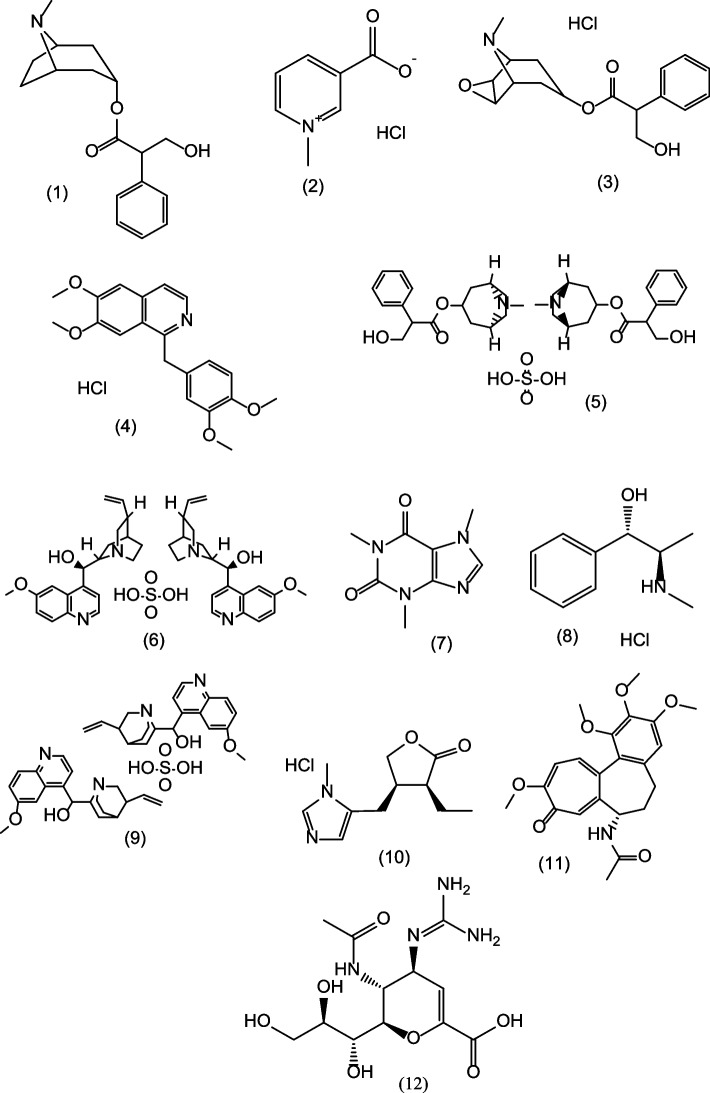
The chemical structures of the tested nitrogenous alkaloids. (1) 1-Hyoscyamine, (2) Trigonelline hydrochloride, (3) (-) Scopolamine hydrochloride, (4) Papaverine hydrochloride, (5) Atropine sulphate, (6) Quinine sulphate, (7) Caffeine, (8) Ephedrine hydrochloride, (9) Quinidine sulphate, (10) Pilocarpine hydrochloride, (11) Colchicine, and (12) Zanamivir

A variety of plant-derived widespread nitrogenous alkaloids (Fig. 1) with various biological activities (Table 1) were selected to further investigate their potential anti-influenza activity.

**Fig. 2:**
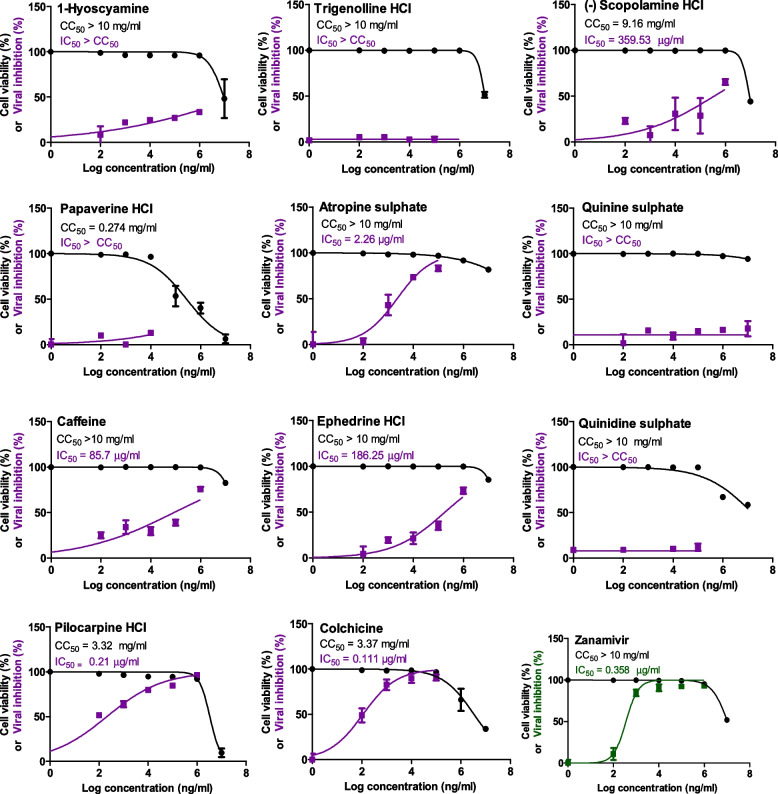
Half-maximal cytotoxic concentrations (CC_50_) in MDCK cells and the half-maximal anti-H5N1 concentrations (IC_50_) for the tested nitrogenous alkaloids (violet) and control zanamivir drug (green). The CC_50_ and IC_50_ of the investigated compounds were assessed by simply plotting log inhibitor versus normalized response (variable slope) and applying the nonlinear regression analyses using GraphPad Prism 5.01 software

First of all, the selected alkaloids were subjected to cytotoxicity investigation in the host MDCK cells. The results of cytotoxicity of the investigated phytochemicals (Fig. 2) curiously revealed that all tested nitrogenous alkaloids have a wide range of non-toxic concentrations in MDCK cells that is exceeding the highest tested concentration for most of them (10 mg/ml).

Defining the toxicity of the tested alkaloids (Table 1) in the MDCK cell line shapes the way for further antiviral bioassays to evaluate their efficacy against both IAV subtypes; A/H5N1 and A/H1N1 in MDCK cells.

Accordingly, all non-toxic concentrations of each compound were used to evaluate the antiviral efficacy of these alkaloids against influenza A/H5N1 virus and to calculate IC_50_ values; in comparison to zanamivir as a control neuraminidase inhibitor. Significantly, atropine sulphate, pilocarpine hydrochloride and colchicine exhibited potent anti-H5N1 activities with extremely low IC_50_ values of 2.300, 0.210 and 0.111 µg/ml, respectively (Fig. 2) and consequently high selectivity indices (SIs) were obtained (Table 2). On the contrary, (-) scopolamine hydrochloride, caffeine and ephedrine hydrochloride showed moderate-to-low anti-H5N1 activities with relatively high IC_50_ values of 359, 85.7 and 186 µg/ml, respectively, compared to the reference NAI drug (IC_50_ = 0.36 µg/ml). However, the rest of the tested alkaloids did not show antiviral potential against the HPAIV virus, thus they were excluded from further antiviral investigations.

**Table 2:** The selectivity index (SI) values for the investigated alkaloids against the tested avian and seasonal influenza A/H5N1 and A/H1N1 viruses

Compound No	Compound Name	Chemical Class	Tested Viruses	CC_50_ (mg/ml)	IC_50_ (µg/ml)	SI
1	1-Hyoscyamine	Tropane Alkaloids	H5N1	> 10	> CC_50_	< 1
			H1N1		ND	ND
**2**	Trigonelline HCl	Pyridine Alkaloids	H5N1	> 10	> CC_50_	< 1
			H1N1		ND	ND
**3**	(-)Scopolamine HCl	Tropane Alkaloids	H5N1	9.16	359	25.51
			H1N1		ND	ND
**4**	Papaverine HCl	Benzyl isoquinoline Alkaloids	H5N1	0.274	> CC_50_	< 1
			H1N1		ND	ND
**5**	Atropine sulphate	Tropane Alkaloids	H5N1	> 10	2.300	> 4,347
			H1N1		0.204	> 49,000
**6**	Quinine sulphate	Quinoline Alkaloids	H5N1	> 10	> CC_50_	< 1
			H1N1		ND	ND
**7**	Caffeine	Xanthine (purine base) Alkaloids	H5N1	> 10	85.7	> 116
			H1N1		ND	ND
**8**	Ephedrine HCl	Amino Alkaloids	H5N1	> 10	186	> 53.76
			H1N1		ND	ND
**9**	Quinidine Sulphate	Quinoline Alkaloids	H5N1	> 10	> CC_50_	< 1
			H1N1		ND	ND
**10**	Pilocarpine HCl	Imidazole Alkaloids	H5N1	3.32	0.210	15,810
			H1N1		0.637	5,212
**11**	Colchicine	Tropolone Alkaloids	H5N1	3.37	0.111	30,360
			H1N1		0.326	10,337
**12**	Zanamivir	NAI (Drug control)	H5N1	> 10	0.360	27,778
			H1N1		0.265	37,736

**Fig. 3:**
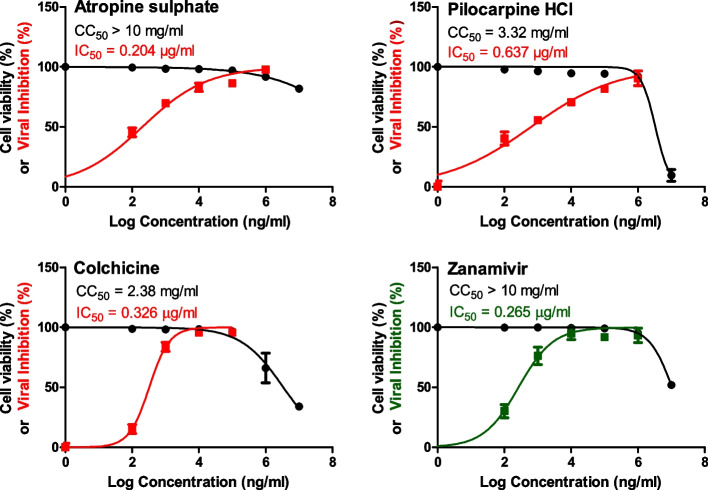
Half-maximal cytotoxic concentrations (CC_50_) in MDCK cells and half-maximal anti-H1N1 concentrations (IC_50_) for the tested nitrogenous alkaloids. The CC_50_ and IC_50_ of the investigated compounds were assessed by simply plotting log inhibitor versus normalized response (variable slope) and applying the nonlinear regression analyses using GraphPad Prism 5.01 software

To evaluate their subtype specificity, the selected alkaloids with potent IC_50_ values (pilocarpine hydrochloride, colchicine and atropine sulphate) were further tested against the seasonal influenza A/H1N1 virus, in comparison to zanamivir as a drug control. Strikingly, atropine sulphate, pilocarpine hydrochloride and colchicine displayed also significant anti-influenza activities against influenza A/H1N1 virus with low IC_50_ values of 0.204, 0.637 and 0.326 µg/ml, respectively, as compared to zanamivir (IC_50_ = 0.265 µg/ml) (Fig. 3).

### Concentration-dependent viral reduction following treatment with atropine sulphate, pilocarpine hydrochloride and colchicine as measured by plaque-reduction assay

**Fig. 4:**
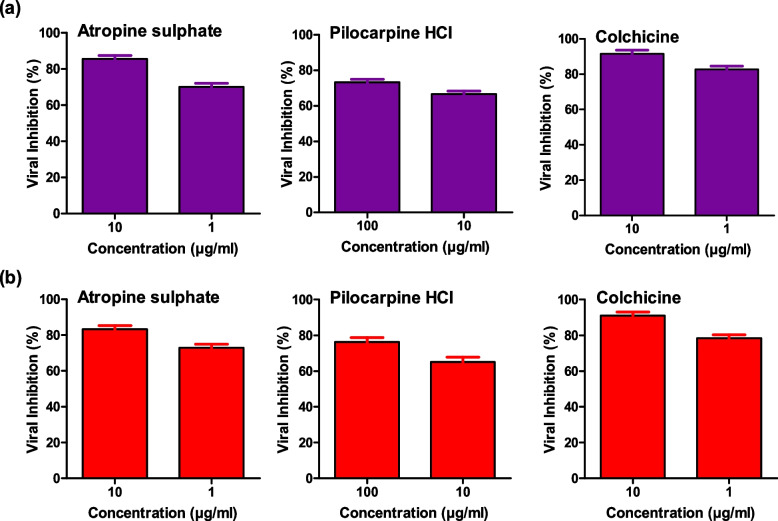
Viral inhibition following treatment with different concentrations of the phytoactive alkaloids as measured by plaque reduction assay. The viral reduction percentage in influenza A/H5N1 (**a**) and A/H1N1 (**b**), following treatment with the three alkaloids, are depicted in violet and red, respectively. Each compound was evaluated independently against both viruses and data were plotted using GraphPad Prism 5.01 software

To further confirm and verify the anti-influenza activity of the three alkaloids against the selected IAVs, the potent alkaloids (according to their low IC_50_ and high SI values) were examined using plaque reduction assay at non-cytotoxic concentrations. Likewise, atropine sulphate, pilocarpine hydrochloride, and colchicine showed a high capacity to reduce viral titers of influenza A/H5N1 and A/H1N1 viruses in MDCK cells at low non-toxic concentrations of the tested alkaloids (Fig. 4). These results confirmed the predefined findings that the three alkaloids can effectively be applied as anti-influenza candidates and nominated them for a further mode of action studies.

### Stage(s) of antiviral action

Three major stages at which the potent alkaloids might reduce the viral titres (virucidal, interference with viral adsorption or viral replication) were investigated. Herein, we investigated if the compound suppresses the viral replication, interferes with viral adsorption into host MDCK cells or works through direct virucidal action on the viral particles away from the host cell. Interestingly, atropine sulphate and pilocarpine hydrochloride could mainly elucidate their anti-influenza efficacy by direct virucidal actions (cell-free status) against the HPAIV (A/H5N1). However, colchicine could primarily reduce the titre of A/H5N1 by interfering with the viral replication mechanisms inside the host cell line (MDCK) (Table 3). Colchicine has also a high ability to inhibit viral adsorption with a percentage of 73% at a concentration of 100 µg/ml.

**Table 3:** Viral inhibition percentage following the investigation of the three possible antiviral stages of action as measured by plaque reduction assay

Compound name	Concentration (µg/ml)	Mechanism of Action		
		**Viral Replication**	**Virucidal**	**Viral Adsorption**
**Atropine sulphate**	1	32.8%	90.5%	36.2%
	10	41.2%	97.6%	45.2%
	100	52.9%	99.99%	52.4%
**Pilocarpine hydrochloride**	10	35.27%	94.88%	37.75%
	100	46.86%	99.07%	44.61%
	1000	53.14%	99.99%	56.86%
**Colchicine**	1	85.7%	25.03%	55.00%
	10	87.00%	38.01%	64.07%
	100	91.08%	47.09%	73.05%

### In silico prediction of possible viral targets

In addition to the previous mechanisms of action, molecular docking studies were carried out for the most active compounds in free forms (atropine, pilocarpine, and colchicine) against promising viral targets to which the two main FDA-approved classes were directed including neuraminidase surface glycoprotein and matrix protein 2 (M2, proton channel). Three active sites have been selected for this study including the influenza A/H1N1 neuraminidase (PDB ID: 6HP0, resolution: 1.88 Å), A/H5N1 neuraminidase (PDB ID: 3CKZ, resolution: 1.90 Å), and proton channel M2 of IAV (PDB ID: 2RLF). The co-crystallized ligands of the three proteins (GJT, zanamivir, and rimantadine, respectively) were used as reference molecules. In the docking studies, we depended on both binding mode (comparing the co-crystallized ligands) and binding energy to investigate the efficiency of binding against the active sites (Table 4).

**Table 4:** Binding free energies (∆G in kcal/mol) of atropine, pilocarpine, colchicine, and the co-crystallized ligands against the active site of influenza A/H1N1 neuraminidase, influenza H5N1 neuraminidase, and influenza M2

Compound name	N1 (H1N1) ∆G [kcal/mol]	N1 (H5N1) ∆G [kcal/mol]	M2 ∆G [kcal/mol]
Atropine	-23.01	-21.81	-12.10
Pilocarpine	-19.00	-16.96	-12.68
Colchicine	-26.13	-25.20	-12.01
GJT The co-crystallized ligand of neuraminidase of H5N1	-24.75	-	-
Zanamivir The co-crystallized ligand of neuraminidase of H5N1	-	-18.87	-
Rimantadine	-	-	-10.49

#### Validation

**Fig. 5:**
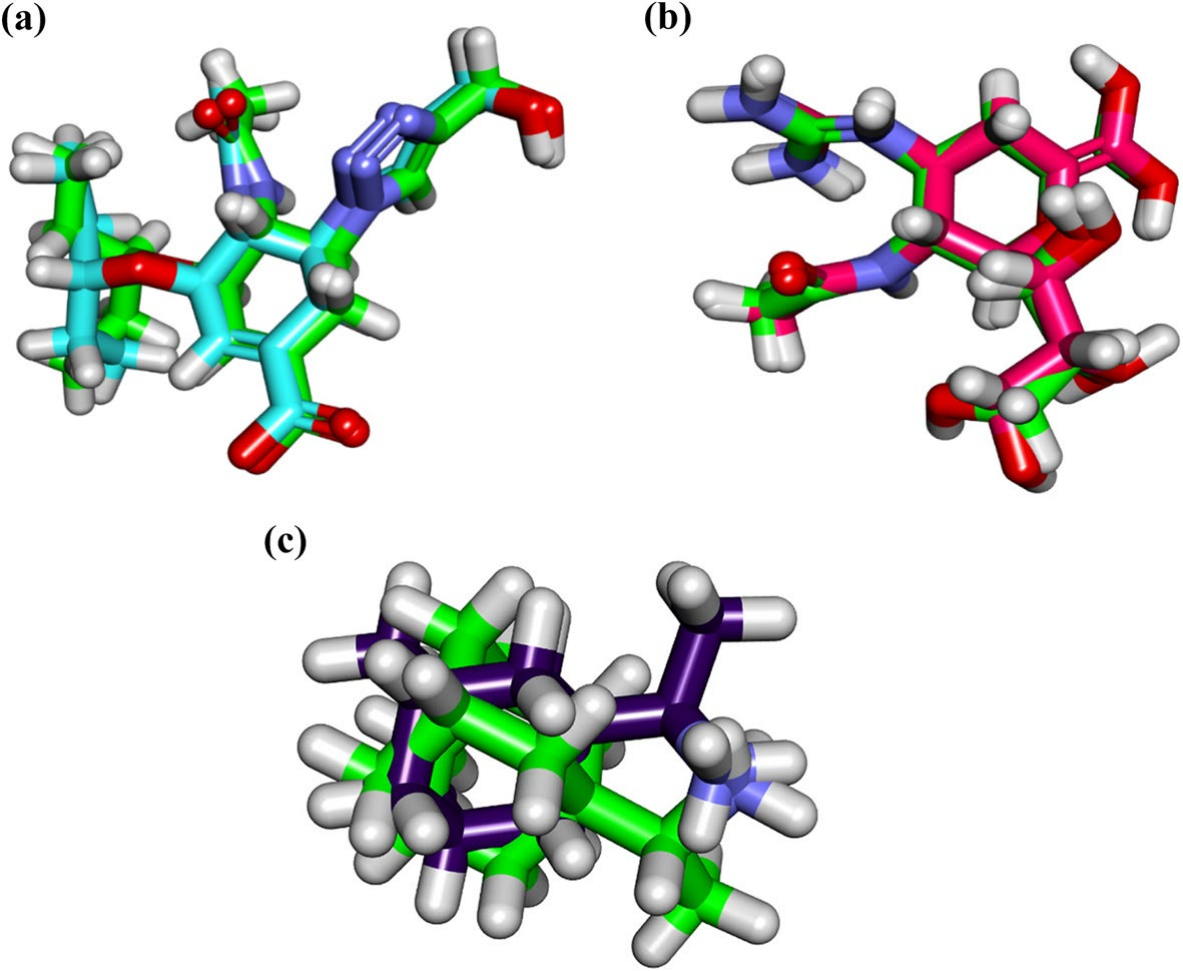
(**a**) Superimposition of the co-crystallized ligand (GJT) of influenza A/H1N1 neuraminidase (carbon atoms in green) and the docked pose of the same ligand (carbon atoms in turquoise). (**b**) Superimposition of the co-crystallized ligand (zanamivir) of influenza H5N1 neuraminidase (carbon atoms in green) and the docked pose of the same ligand (carbon atoms in pink). (**c**) Superimposition of the co-crystallized ligand (rimantadine) of influenza M2 (carbon atoms in green) and the docked pose of the same ligand (carbon atoms in violet)

Re-docking the co-crystallized ligands in the active sites of N1 neuraminidase subtype from influenza A/H1N1 and influenza A/H5N1 viruses as well as the IAV M2 protein utilizing MMFF94X as a force field and ASE as a scoring function allowed for the protocol's validation. The small RMSD values between the docked poses and the co-crystallized ligands during the validation step (0.59, 0.44, and 1.58 Å for the influenza A/H1N1-type, A/H5N1-type neuraminidase, and influenza conserved M2 proton channel protein, respectively) indicated the feasibility of the used methodology for the intended docking experiments. Additionally, accessing the reported binding mode of co-crystallized ligands using the docking technique supported the choice of the docking algorithm (Fig. 5a-c).

#### Docking studies against influenza H1N1 neuraminidase

**Fig. 6:**
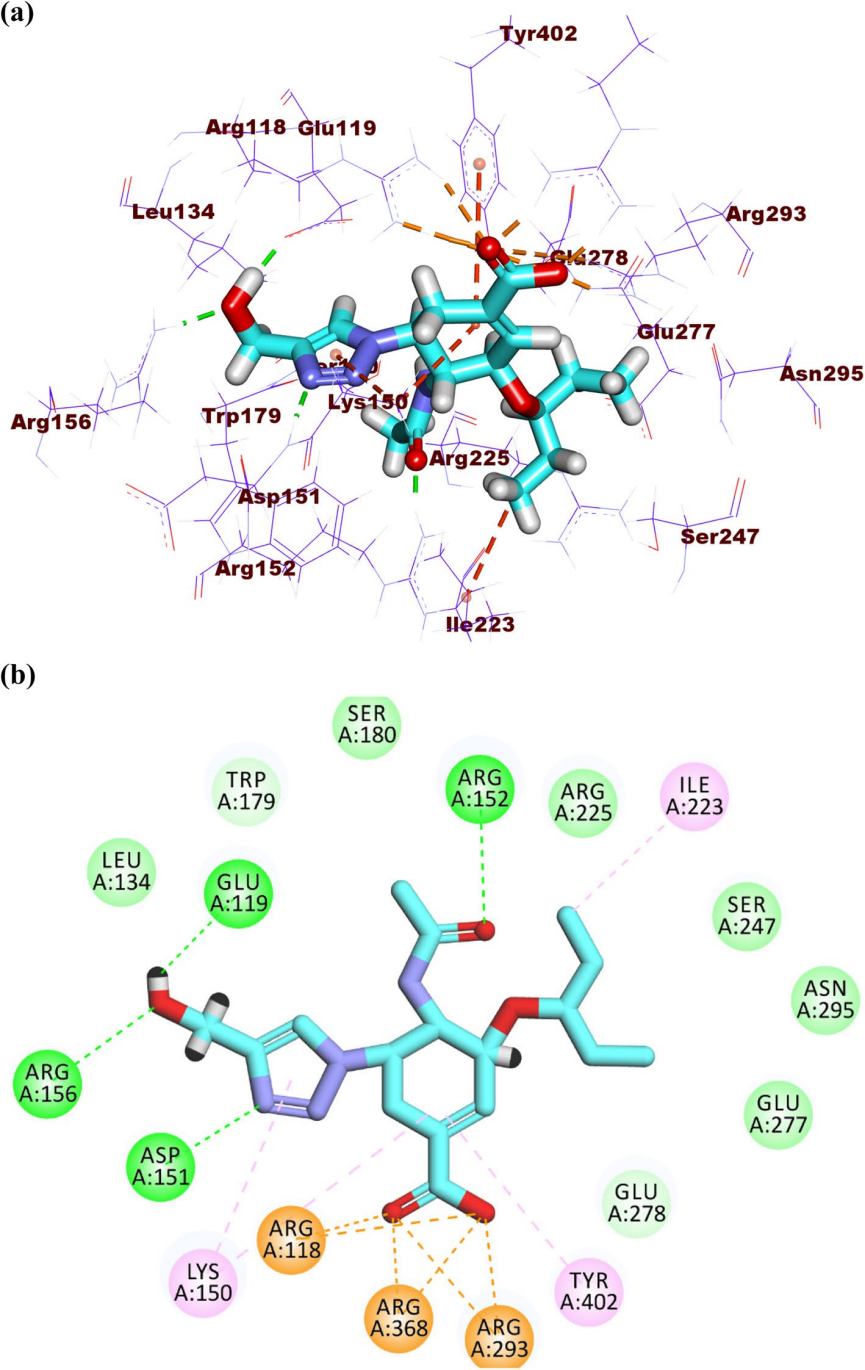
(**a**) 3D and (**b**) 2D of GJT in the active site of influenza A/H1N1 neuraminidase

The co-crystalized ligand (GJT) showed a binding sore of -24.75 kcal/mol against influenza A/H1N1 neuraminidase. The (1H-1,2,3-triazol-4-yl)methanol moiety occupied the first pocket of the active site forming three hydrogen bonds with Glu119, Arg156 and Asp151. Moreover, it formed one hydrogen bond with Lys150. The cyclohex-1-ene-1-carboxylic acid moiety occupied the second pocket forming six electrostatic attractions with Arg293, Arg368, and Arg118. Also, it formed two hydrophobic attractions with Arg118 and Tyr402. Furthermore, the 2-oxopropyl and pentan-3-yloxy moieties occupied the third pocket forming one hydrogen and one hydrophobic bond with Arg152 and Ile223, respectively (Fig. 6a-b).

**Fig. 7:**
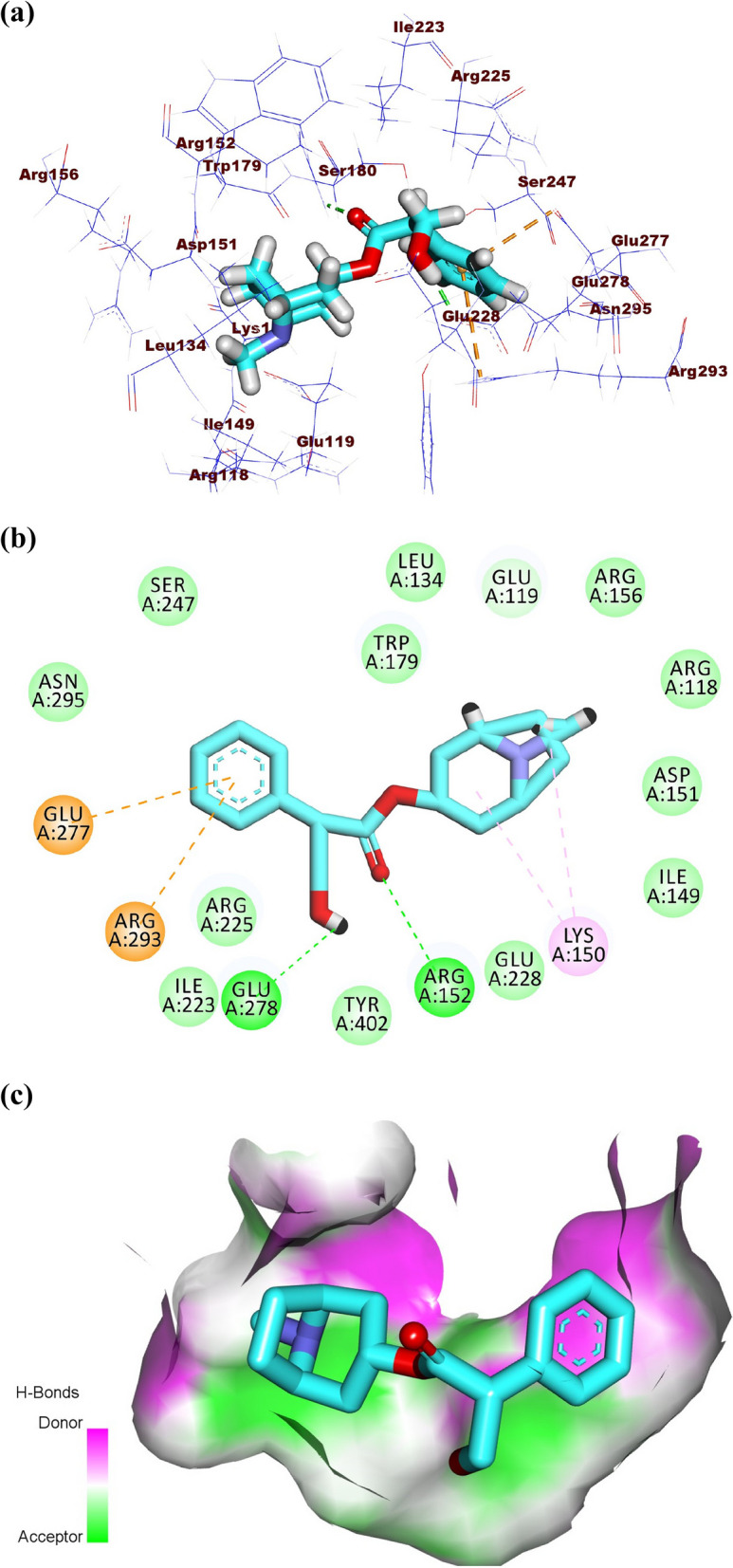
(**a**) 3D, (**b**) 2D and (**c**) Surface map of atropine in the active site of influenza A/H1N1 neuraminidase

The tested molecules interacted with the active site of influenza A/H1N1 neuraminidase showing binding modes almost the same as that of GJT. Atropine exhibited a binding affinity of -23.01 kcal/mol against influenza A/H1N1 neuraminidase. In detail, the phenyl moiety occupied the first pocket of the active site forming an electrostatic attraction with Glu277 and Arg293. The 8-methyl-8-azabicyclo[3.2.1]octane occupied the second pocket in close contact with Arg118, Trp179, Leu134, Glu119, Arg156, Asp151, Ile149, and Glu228. The 4-hydroxybutan-2-one linker moiety occupied the third pocket forming two hydrogen bonds with Arg152 and Glu278 (Fig. 7a-c).

**Fig. 8:**
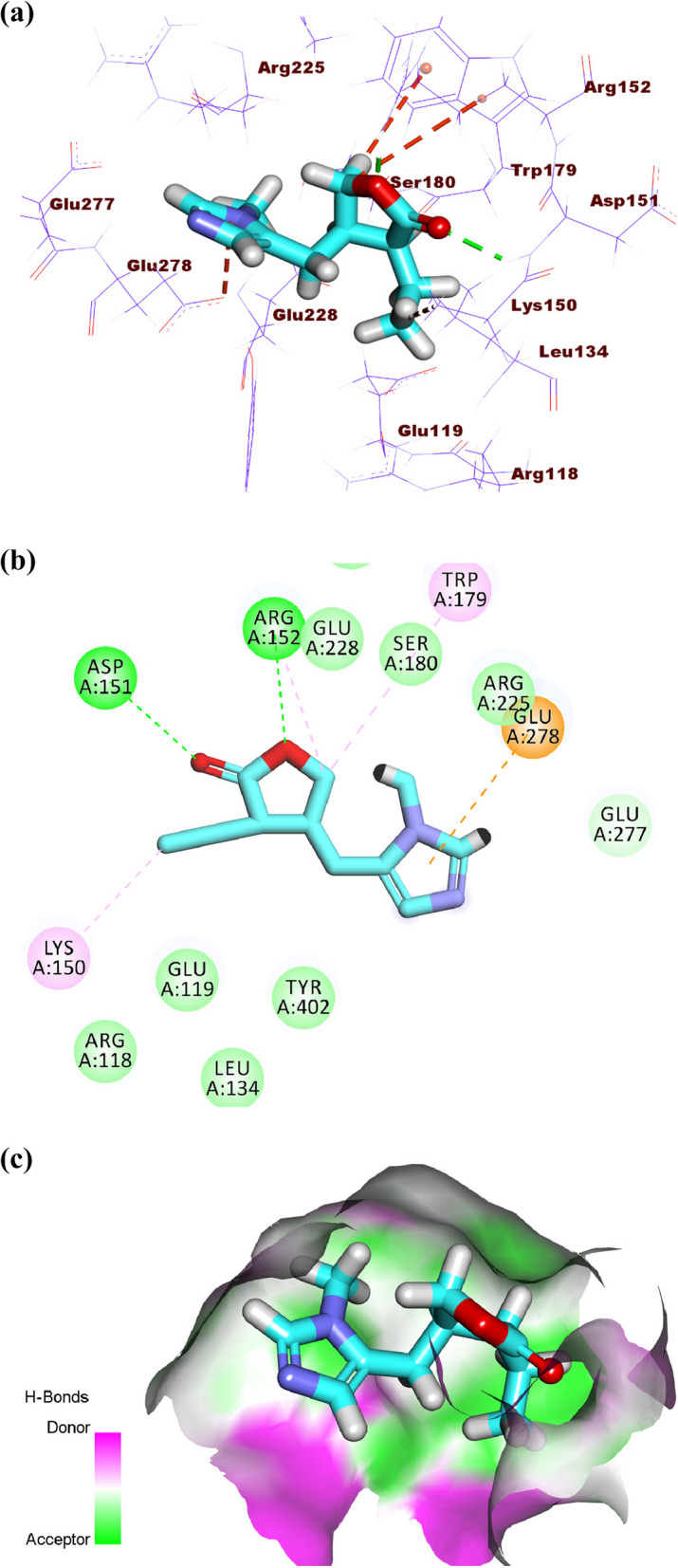
(**a**) 3D, (**b**) 2D and (**c**) Surface map of pilocarpine in the active site of influenza A/H1N1 neuraminidase

Pilocarpine showed a binding score of -19.00 kcal/mol against influenza A/H1N1 neuraminidase. The (S)-3-ethyldihydrofuran-2(3*H*)-one moiety occupied the first pocket of the active site forming two hydrogen bonds with Arg152 and Asp151. Also, it formed three hydrophobic interactions with Trp179, Arg152, and Lys150. The 1-methyl-1H-imidazole moiety occupied the second pocket forming one electrostatic attraction with Glu278 (Fig. 8a-c).

**Fig. 9:**
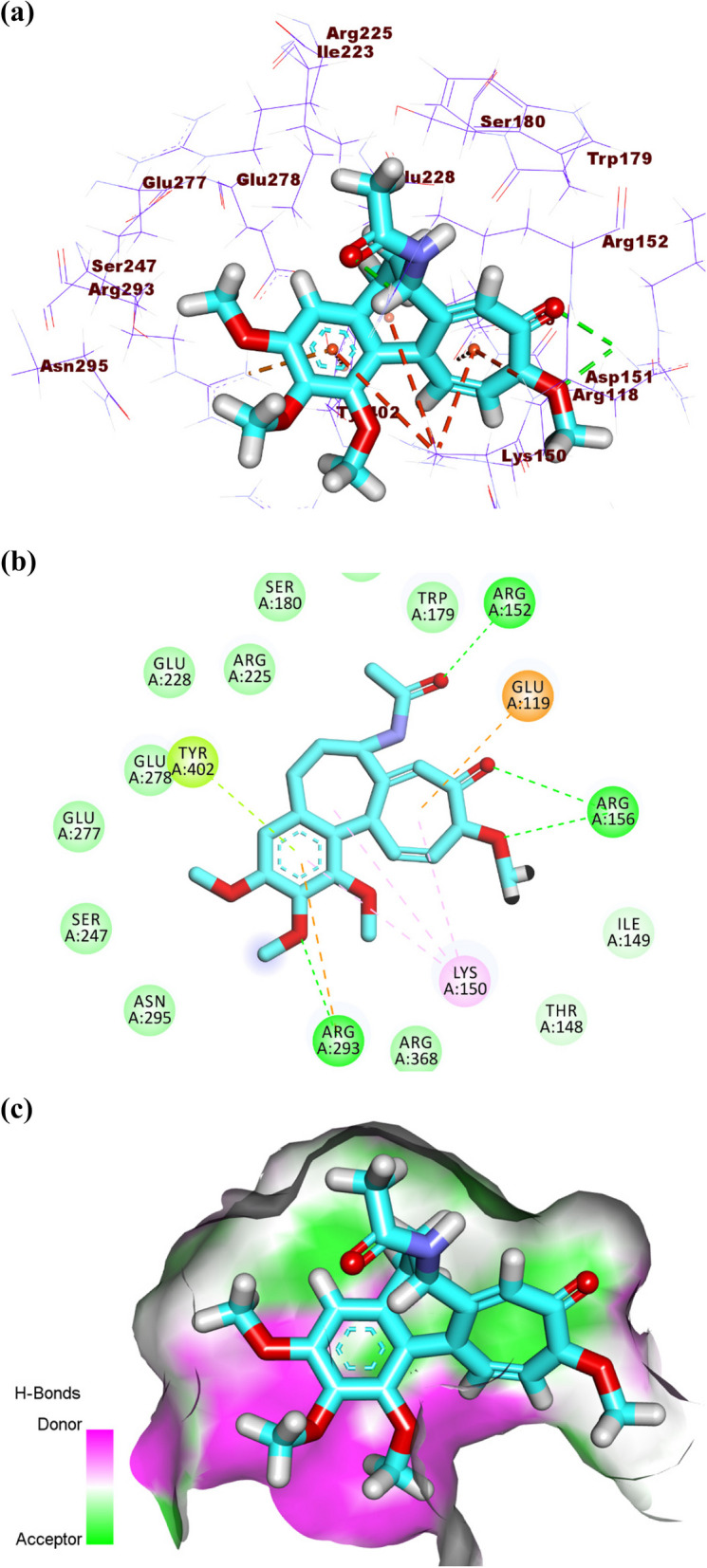
(**a**) 3D, (**b**) 2D, and (**c**) Surface map of colchicine in the active site of influenza A/H1N1 neuraminidase

Colchicine produced a binding energy of -26.13 kcal/mol against influenza A/H1N1 neuraminidase. The tropolone moiety occupied the first pocket of the active site forming two hydrogen bonds with Arg156 and one electrostatic bond with Glu119. Also, it formed one hydrophobic interaction with Lys150. The acetamide moiety occupied the second pocket forming one hydrogen bond with Arg152. The 1,2,3-trimethoxybenzene occupied the third pocket forming one hydrogen bond and one electrostatic attraction with Arg293. In addition, it formed two hydrophobic attractions with Lys150 and Tyr402 (Fig. 9a-c).

#### Docking studies against influenza A/H5N1 neuraminidase

**Fig. 10:**
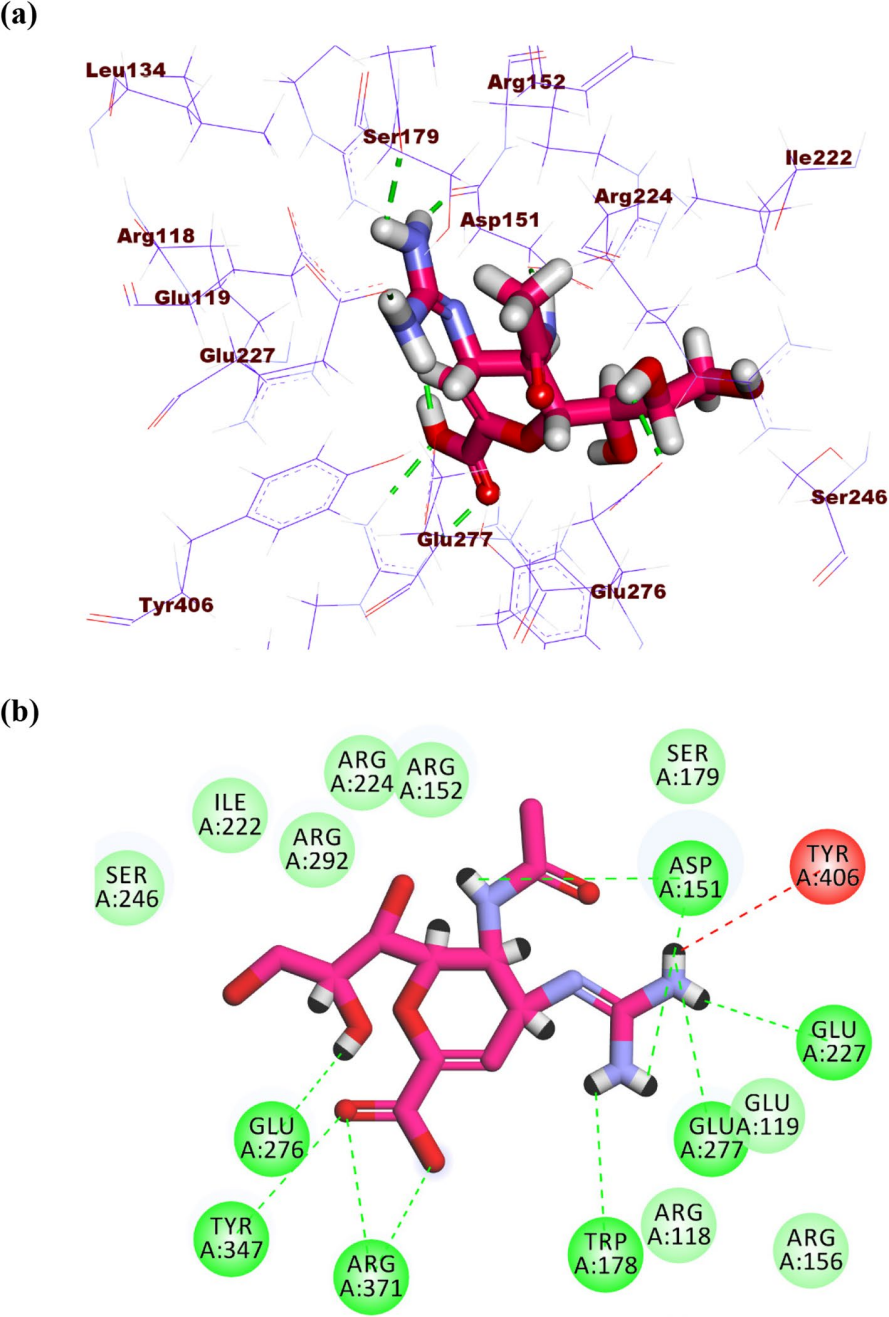
(**a**) 3D, and (**b**) 2D of zanamivir in the active site of influenza A/H5N1 neuraminidase

Starting with the co-crystalized ligand (zanamivir), it showed a binding sore of -18.87 kcal/mol against influenza A/H5N1 neuraminidase. The guanidine and acetamide moieties were oriented into the first pocket of the active site forming five hydrogen bonds with Glu277, Trp178, Glu,227 and Asp151. In addition, it formed one electrostatic attraction with Tyr406. The cyclohex-1-ene-1-carboxylic acid moiety was oriented into the second pocket forming three hydrogen bonds with Arg371, Tyr347, and Glu276. Furthermore, the propane-1,2,3-triol moiety occupied the third pocket forming one hydrogen bond with Glu276 (Fig. 10a-b).

**Fig. 11:**
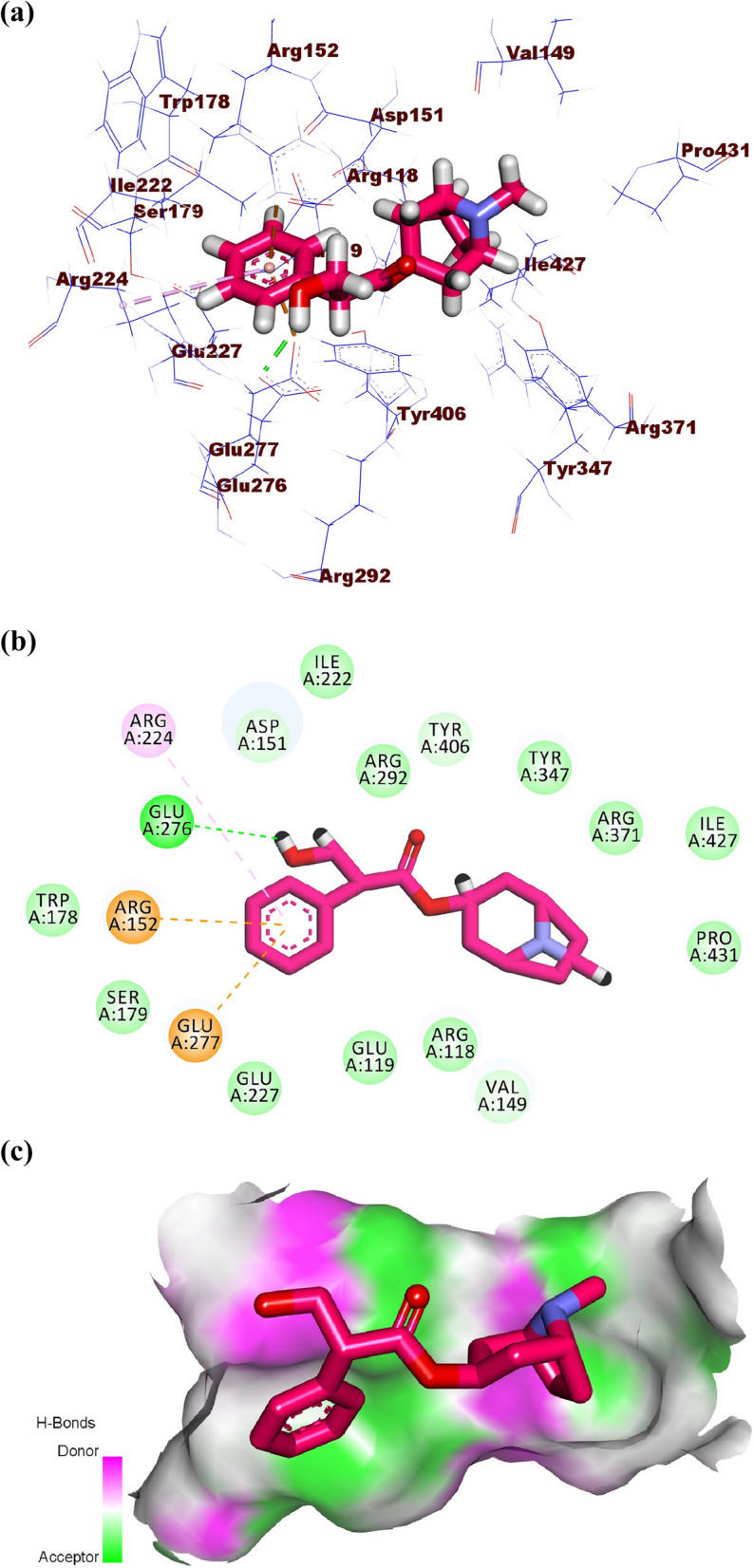
(**a**) 3D, (**b**) 2D, and (**c**) Surface map of atropine in the active site of influenza A/H5N1 neuraminidase

Atropine, pilocarpine, and colchicine showed efficient binding in the active site of influenza H5N1 neuraminidase. These compounds exhibited binding modes like that of zanamivir. Atropine exhibited a binding affinity of -21.81 kcal/mol against influenza H5N1 neuraminidase. The phenyl moiety occupied the first pocket of the active site forming two electrostatic attractions with Glu277 and Arg152. In addition, it formed one hydrophobic interaction with Arg224. The 8-methyl-8-azabicyclo [3.2.1]octane occupied the second pocket in close contact with Arg118, Thr439, Arg371, Val149, Tyr347, pro431, Ile427, and Glu119. The 4-hydroxybutan-2-one linker moiety occupied the third pocket forming one hydrogen bond with Glu276 in close contact with Asp151, Arg292, Ile222, and Tyr406 (Fig. 11a-c).

**Fig. 12:**
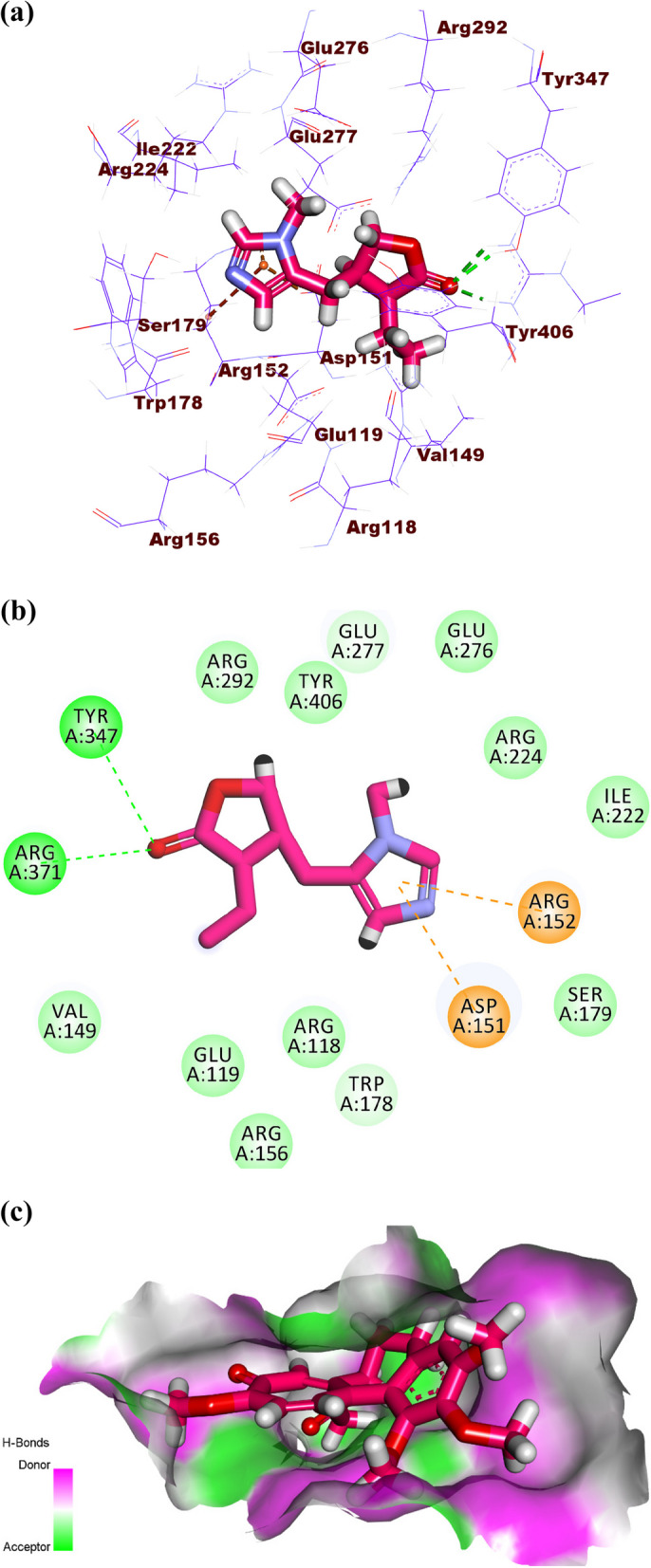
(**a**) 3D, (**b**) 2D, and (**c**) Surface map of pilocarpine in the active site of influenza A/H5N1 neuraminidase

Pilocarpine showed a binding score of -16.96 kcal/mol against influenza A/H5N1 neuraminidase. The 1-methyl-1H-imidazole moiety occupied the first pocket forming two electrostatic attractions with Arg152 and Asp151. Moreover, the (S)-3-ethyldihydrofuran-2(3*H*)-one moiety occupied the second pocket of the active site forming two hydrogen bonds with Tyr347 and Arg371 (Fig. 12a-c).

**Fig. 13:**
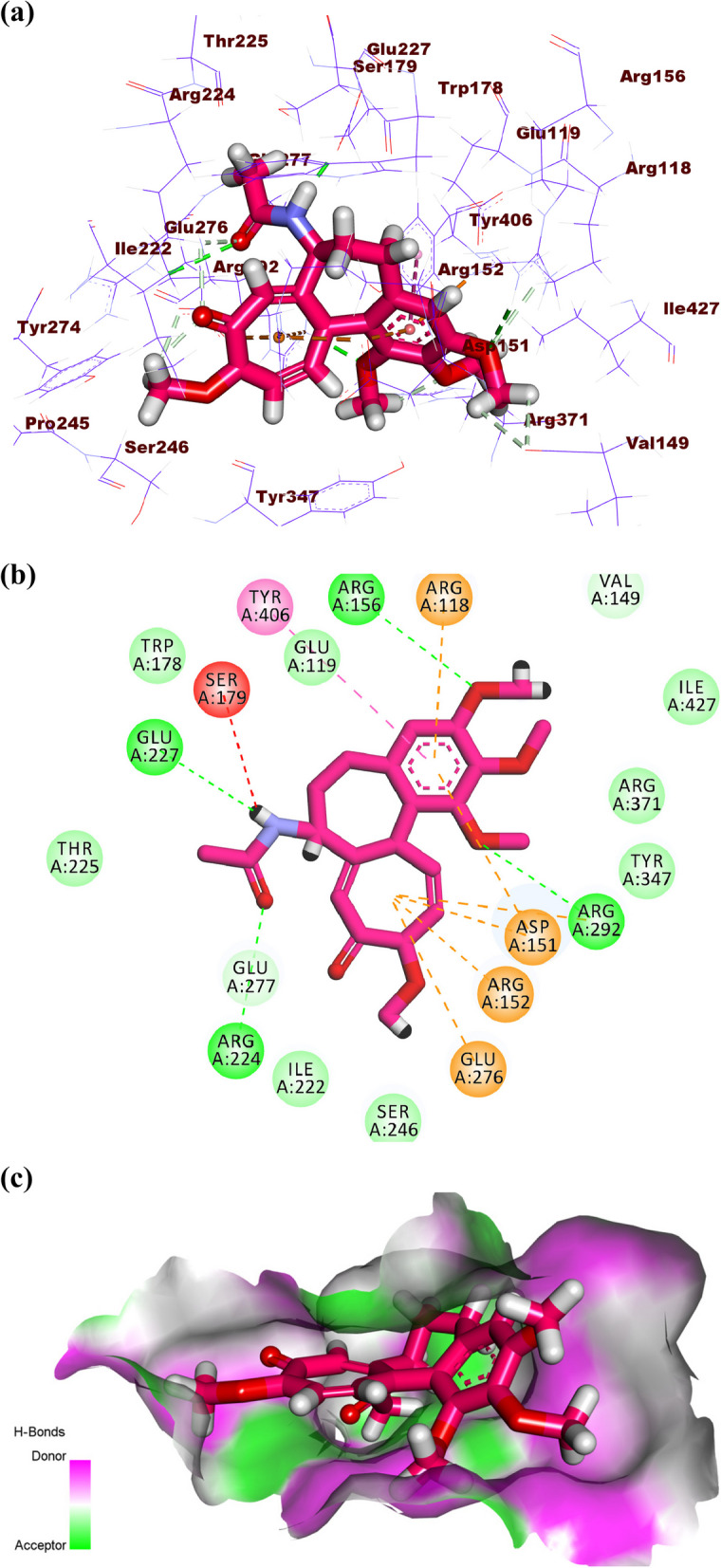
(**a**) 3D, (**b**) 2D and (**c**) Surface map of colchicine docked into the active site of influenza A/H5N1 neuraminidase

Colchicine produced a binding energy of -25.20 kcal/mol against influenza H5N1 neuraminidase. The tropolone moiety occupied the first pocket of the active site forming four electrostatic interactions with Asp151, Arg152, and AGlu276. The 1,2,3-trimethoxybenzene occupied the second pocket forming two hydrogen bonds with Arg292 and Arg156. Also, it formed two electrostatic attractions with Asp151 and Arg118. In addition, it formed one hydrophobic attraction with Tyr406. The acetamide moiety occupied the third pocket forming two hydrogen bonds with Glu227 and Arg224. Additionally, it formed one electrostatic interaction with Ser179 (Fig. 13a-c).

#### Docking studies against proton channel M2 of IAV

**Fig. 14:**
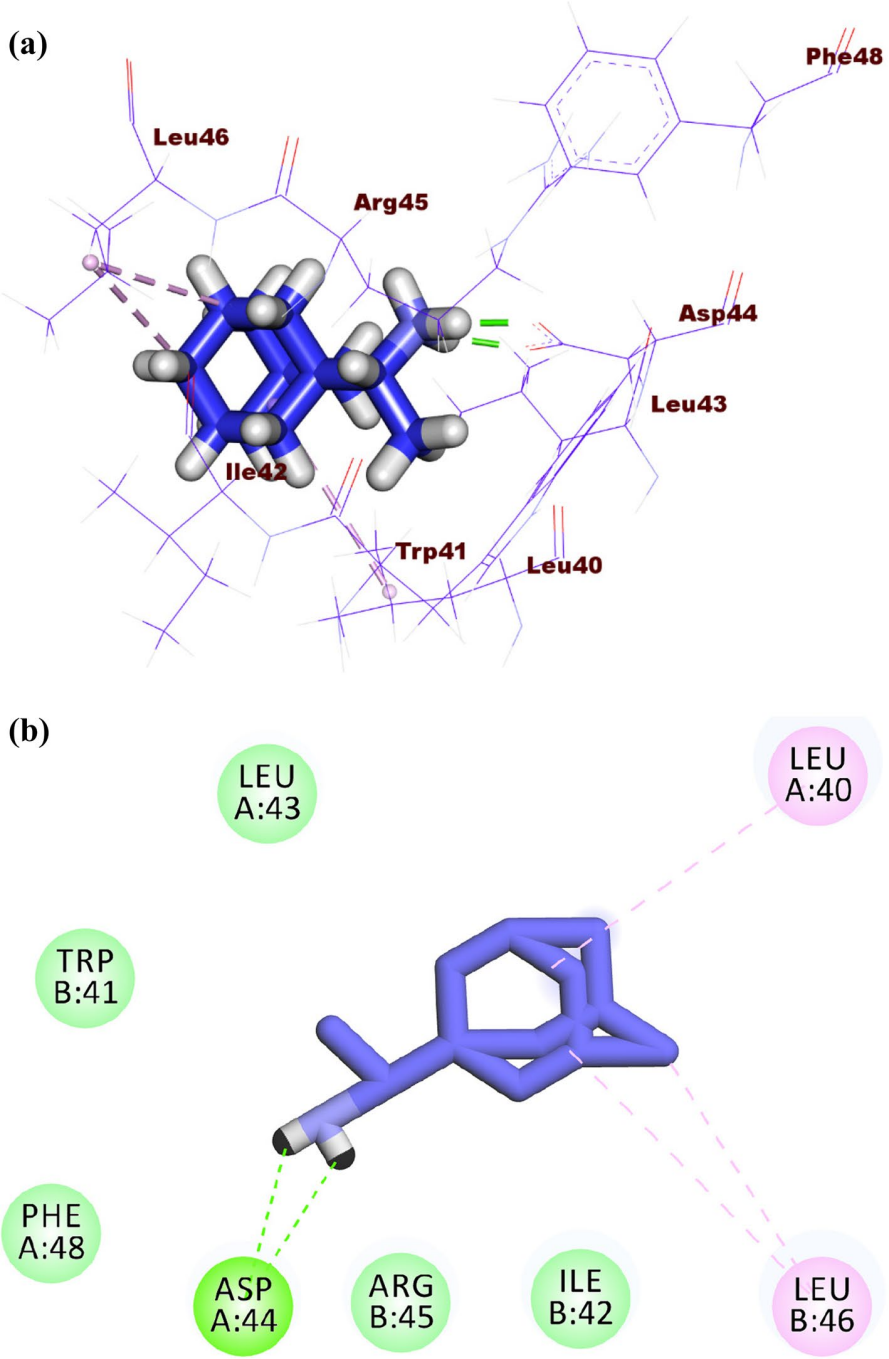
(**a**) 3D, and (**b**) 2D of rimantadine in the active site of IAV M2 proton channel

The co-crystalized ligand (rimantadine) showed a binding sore of -10.49 kcal/mol against IAV M2 protein. The ethanamine moiety was oriented into the deep pocket of the receptor forming two hydrogen bonds with Asp44. The adamantane moiety was oriented into the outer region of the active site forming three hydrophobic interactions with Leu46 and Leu40 (Fig. 14a-b).

The binding modes tested compounds (atropine, pilocarpine, and colchicine) were investigated. Although atropine and pilocarpine showed good binding free energies, both of them failed to have a correct binding mode with the active site of the IAV M2 proton channel. On the other hand, colchicine exhibited an excellent binding mode. Accordingly, the binding pattern of colchicine was discussed as follows.

**Fig. 15:**
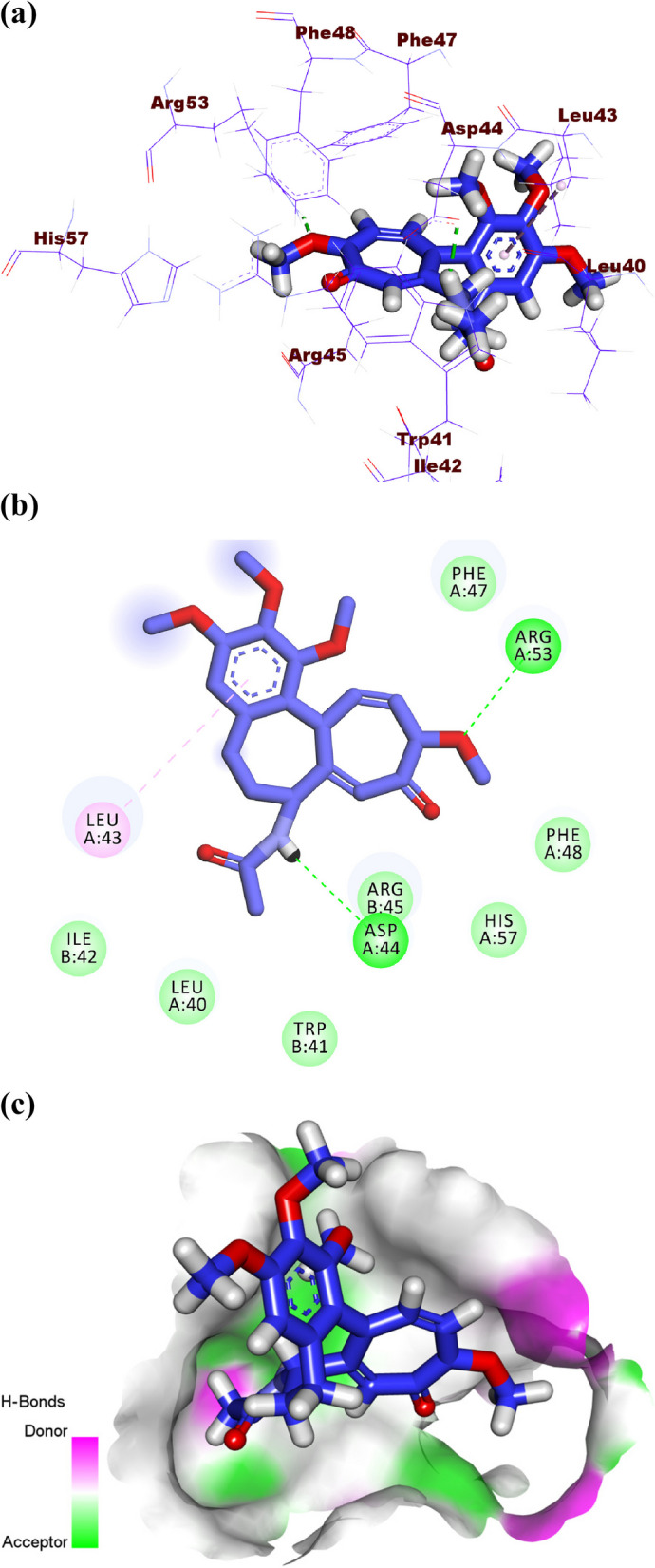
(**a**) 3D, (**b**) 2D, and (**c**) Surface map of colchicine docked into the active site of IAV M2 proton channel

Colchicine produced a binding energy of -12.01 kcal/mol against the IAV M2 proton channel. The acetamide moiety occupied the inner pocket of the active site forming one hydrogen bond with Asp44. The tropolone and 1,2,3-trimethoxybenzene moieties were oriented into the outer region of the active site forming one hydrogen bond with Arg53 and one hydrophobic bond with Leu43 (Fig. 15a-c).

## Discussion

Influenza pandemics and seasonal epidemics threaten the public health of human and animal populations [68]. Annually, seasonal influenza epidemics result in about 3–5 million cases of severe illness, and about 290,000 to 650,000 respiratory deaths [69]. Seasonal influenza vaccines and limited options of anti-influenza medications are currently available, however, their effectiveness has always been debated due to the emergence of resistance to antivirals and relatively low and unpredictable efficiency of the seasonal influenza vaccines compared to other vaccines [70]. The classical M_2_ ion channel blockers (rimantadine and amantadine) and neuraminidase inhibitors (oseltamivir and zanamivir) are two classes of antiviral medications that have been authorized by the FDA organization for the treatment of influenza [71, 72]. However, misuse of these therapies and the continuous genetic and antigenic drift of IAVs during replication in different host species has resulted in drug-resistant and/or reassortant strains [73], with high risk to public health [73–75]. Consequently, the global fear of future influenza pandemics urges the need to innovate broad-spectrum anti-influenza medications that are not subtype- or strain-specific [76]. Phytomedicine is one of the most effective ways to cure various ailments [21]. In this regard, we aimed in this study to determine the anti-influenza potential of naturally available nitrogenous alkaloids against two distinct IAV subtypes, influenza A/H5N1 and A/H1N1.

Notably, non-cytotoxic concentrations of the tropane alkaloid, atropine sulphate, exerted anti-influenza effects against the avian A/H5N1 virus and the seasonal A/H1N1 virus with high selectivity indices through direct virucidal action against the avian virus subtype of H5N1 subtype. However, previous studies regarding the anti-influenza activities of atropine sulphate are rare. Prior investigations on atropine revealed its in vitro antiviral activities against herpes simplex virus type-1 (HSV-1) and parainfluenza type-3 (PI-3) with therapeutic doses between 0.05 and 0.8 µg/ml [39].

Likewise, our results demonstrated that pilocarpine hydrochloride exhibits a promising anti-influenza efficacy against both influenzas A viruses, subtypes H5N1 and A/H1N1, in a concentration-dependent manner with potent IC_50_ values and high SI values when compared to the FDA-approved anti-influenza reference drug used in our study (zanamivir). It has been proven to counteract the influenza A/H5N1 virus via cell-free direct virucidal action. In literature, rare or no studies were reported on the antiviral effects of pilocarpine hydrochloride in medical uses, providing that it was known to be used in ophthalmic solutions to treat glaucoma for many years [77].

Significant anti-influenza effects have also been exerted by the tropolone alkaloid, colchicine, against both IAVs subtypes and showed also to interfere with the viral replication of the avian influenza A/H5N1 virus. From the toxicological background, our findings suggested that the IC_50_ values of colchicine when tested against both tested IAVs, influenza A/H5N1 and A/H1N1 virues, were 0.076 and 0.65 µg/ml, respectively and that is far below the toxic range described earlier (0.6 mg) [78]. Concerning the anti-influenza effects of colchicine, no information was available regarding this area of study. Prior studies proved that colchicine is an antimitotic agent and this may aid in understanding the mechanism by which it affects the H5N1 viral replication [79].

In previous investigations, papaverine has been proven to exert anti-influenza activities against different strains of IAVs and parainfluenza viruses with IC_50_ values ranging between 2.02 and 36.41 µM [43]. Nonetheless, in the primary screening stage of our study, papaverine hydrochloride showed poor anti-influenza activity against the highly pathogenic avian A/H5N1 virus. However, quinine sulphate has been described earlier to exert an antiviral effect against IAV (A/Puerto Rico/8/1934(H1N1) in mice [47], it elucidated no anti-influenza activity against influenza A/H5N1 virus in our findings. Contextually, a previous study regarding the anti-influenza potential of L-Ephedrine against A/PR8/34 (H1N1 virus) proved its efficacy against the tested viruses with EC_50_ values ranging from 5.66 to 10.96 µg/ml [55]. Our findings proved poor anti-influenza activity of ephedrine hydrochloride against avian influenza A/H5N1 virus with an IC_50_ value of 186.25 µg/ml when compared to the reference zanamivir drug. The rest of the tested alkaloids showed poor or no anti-influenza activity during the primary screening using the HPAIV A/H5N1.

In the computational studies, the three most active alkaloids (atropine, pilocarpine, and colchicine) were subjected to molecular docking investigations against three viral proteins. The first and second proteins are the neuraminidases influenza H1N1, and influenza H5N1, respectively. The neuraminidases (sialidases) bind to the sialic acid receptors in the cell surface causing the cleaving of the receptor and allowing the virus release outside the host cell to infect other cells [80]. Accordingly, it was selected to figure out another mechanism of action for the selected compounds. Fortunately, the tested compounds showed similar binding modes to that of the reference molecules (the co-crystallized ligands) with slight variation for each tested molecule. For the binding energy, it was found that colchicine has the highest energy of binding indicating higher affinity against the two targets than atropine and pilocarpine. Additionally, atropine showed higher affinity than pilocarpine. Regarding the influenza M2 protein, it has an essential role in viral adsorption as it is responsible for the equilibration of the pH across the membrane of the virus to facilitate cell entry in addition to the entry across the Golgi membrane viral maturation [81]. Interestingly, the in vitro and the in silico results were consistent. In the in vitro assay, colchicine expressed also a high ability to inhibit viral adsorption with a percentage of 73% at a concentration of 100 µg/ml while both atropine and pilocarpine were not able to inhibit viral adsorption. The computational studies showed that colchicine exhibited a correct binding mode against the influenza M2 protein while both atropine and pilocarpine failed to show correct binding modes.

Ultimately, this study suggests the anti-influenza efficacy of three nitrogenous alkaloids; namely atropine sulphate, pilocarpine hydrochloride and colchicine. Nevertheless, further studies must be conducted to validate the in vivo bioavailability and efficacy of the three alkaloids against influenza virus infections.

## Conclusion

Since the emergence of the devastating COVID-19 pandemic, drug repurposing as an accelerated approach to identify new antiviral indications of commercially available FDA approved drugs attracted more attention and contributed actively to control the infection. Due to their various pharmacological effects, including antiviral activities, nitrogenous alkaloids are a major class of phytochemicals that are grasping the attention of many antiviral researchers. The anti-influenza efficacy of various biologically active alkaloids against avian IAV seasonal human IAV could be successfully investigated in this work. Significantly, atropine sulphate, pilocarpine hydrochloride and colchicine showed substantial anti-influenza effects against the designated strains. Additionally, atropine, pilocarpine, and colchicine showed excellent in silico potentialities to bind and inhibit the neuraminidases of both influenza H1N1, and influenza H5N1. Also, in agreement with in vitro results, only colchicine could bind correctly against proton channel M2 of IAV.

## Data Availability

All data generated or analyzed during this study are included in this published article.

## Abbreviations

COVID-19: Coronavirus disease 2019
IC_50_: Half-maximal inhibitory concentration
EC_50_: Half-maximal effective cytotoxic concentration
CC_50_: Half-maximal cytotoxic concentration
HPAIV: Highly pathogenic avian influenza virus
IAV: Influenza A virus
FDA: Food and drug administration

## References

[1] Gaitonde DY, Moore FC, Morgan MK (2019). Influenza: diagnosis and treatment. Am Fam Phys..

[2] Lo CY, Tang YS, Shaw PC (2018). Structure and function of Influenza virus ribonucleoprotein. Virus Protein Nucleoprotein Compl..

[3] Collin EA, Sheng Z, Lang Y, Ma W, Hause BM, Li F (2015). Cocirculation of two distinct genetic and antigenic lineages of proposed Influenza D virus in cattle. J Virol.

[4] Horimoto T, Kawaoka Y (2005). Influenza: lessons from past pandemics, warnings from current incidents. Nat Rev Microbiol.

[5] Ding Y, Dou J, Teng Z, Yu J, Wang T, Lu N, Wang H, Zhou C (2014). Antiviral activity of baicalin against influenza A (H1N1/H3N2) virus in cell culture and in mice and its inhibition of neuraminidase. Arch Virol.

[6] King PT, Londrigan SL (2021). The 1918 influenza and COVID-19 pandemics: the effect of age on outcomes. Respirol (Carlton Vic).

[7] WHO: COVID-19 weekly epidemiological update, edition 110, 21 September 2022. 2022. Available online at https://www.who.int/publications/m/item/weekly-epidemiological-update-on-covid-19---21-september-2022. Accessed 15 Nov 2023.

[8] Mostafa A, Abdelwhab EM, Mettenleiter TC, Pleschka S (2018). Zoonotic potential of influenza A viruses: a comprehensive overview. Viruses.

[9] Nguyen LT, Stevenson MA, Firestone SM, Sims LD, Chu DH, Van Nguyen L, Nguyen TN, Le KT, Isoda N, Matsuno K (2020). Spatiotemporal and risk analysis of H5 highly pathogenic avian influenza in Vietnam, 2014–2017. Prev Vet Med.

[10] Sealy JE, Fournie G, Trang PH, Dang NH, Sadeyen JR, Thanh TL, van Doorn HR, Bryant JE, Iqbal M (2019). Poultry trading behaviours in Vietnamese live bird markets as risk factors for avian influenza infection in chickens. Transbound Emerg Dis.

[11] Ulyanova V, Shah Mahmud R, Laikov A, Dudkina E, Markelova M, Mostafa A, Pleschka S, Ilinskaya O (2020). Anti-influenza activity of the ribonuclease binase: Cellular targets detected by quantitative proteomics. Int J Mol Sci.

[12] Osterholm MT, Kelley NS, Sommer A, Belongia EA (2012). Efficacy and effectiveness of Influenza vaccines: a systematic review and meta-analysis. Lancet Infect Dis.

[13] El-Shesheny R, Bagato O, Kandeil A, Mostafa A, Mahmoud SH, Hassanneen HM, Webby RJ, Ali MA, Kayali G (2016). Re-emergence of amantadine-resistant variants among highly pathogenic avian influenza H5N1 viruses in Egypt. Infect Genet Evol.

[14] Choi J-G, Kim YS, Kim JH, Chung H-S (2019). Antiviral activity of ethanol extract of Geranii Herba and its components against Influenza viruses via neuraminidase inhibition. Sci Rep.

[15] Zhang S, Tian H, Cui J, Xiao J, Wang M, Hu Y (2016). The c-Jun N-terminal kinase (JNK) is involved in H5N1 influenza a virus RNA and protein synthesis. Arch Virol.

[16] Cetina-Montejo L, Ayora-Talavera G, Borges-Argáez R (2019). Zeylanone epoxide isolated from diospyros anisandra stem bark inhibits influenza virus in vitro. Arch Virol.

[17] Tran TT, Kim M, Jang Y, Lee HW, Nguyen HT, Nguyen TN, Park HW, Le Dang Q, Kim J-C (2017). Characterization and mechanisms of anti-influenza virus metabolites isolated from the Vietnamese medicinal plant polygonum chinense. BMC Complement Altern Med.

[18] Mehrbod P, Ebrahimi SN, Fotouhi F, Eskandari F, Eloff JN, McGaw LJ, Fasina FO (2019). Experimental validation and computational modeling of anti-influenza effects of quercetin-3-O-α-L-rhamnopyranoside from indigenous South African medicinal plant Rapanea melanophloeos. BMC Complement Altern Med.

[19] Watanabe K, Rahmasari R, Matsunaga A, Haruyama T, Kobayashi N (2014). Anti-influenza viral effects of honey in vitro: potent high activity of manuka honey. Arch Med Res.

[20] Ben-Shabat S, Yarmolinsky L, Porat D, Dahan A (2020). Antiviral effect of phytochemicals from medicinal plants: applications and drug delivery strategies. Drug Deliv Transl Res.

[21] Lenz E, Müller C, Mostafa A, Dzieciolowski J, Kanrai P, Dam S, Cwientzek U, Prenner L-N, Pleschka S (2018). Authorised medicinal product Aspecton® oral drops containing thyme extract KMTv24497 shows antiviral activity against viruses which cause respiratory infections. J Herb Med.

[22] Amirkia V, Heinrich M (2014). Alkaloids as drug leads–A predictive structural and biodiversity-based analysis. Phytochem Lett.

[23] Harborne JB. Textbook of Phytochemical Methods. A Guide to Modern Techniques of Plant Analysis. 5th Edition. London: Chapman and Hall Ltd; 1998. p. 21–72.

[24] Lu JJ, Bao JL, Chen XP, Huang M, Wang YT (2012). Alkaloids isolated from natural herbs as the anticancer agents. Evid-based Complement Altern Med.

[25] Dey P, Kundu A, Kumar A, Gupta M, Lee BM, Bhakta T, Dash S, Kim HS (2020). Analysis of alkaloids (indole alkaloids, isoquinoline alkaloids, tropane alkaloids). Recent advances in natural products analysis.

[26] Shi Z, Zou W, Zhu Z, Xiong Z, Li S, Dong P, Zhu Z (2022). Tropane alkaloids (hyoscyamine, scopolamine and atropine) from genus Datura: extractions, contents, syntheses and effects. Ind Crops Prod.

[27] Roy H, Nandi S (2019). In-silico modeling in drug metabolism and interaction: current strategies of lead discovery. Curr Pharm Design.

[28] Eissa IH, Ibrahim MK, Metwaly AM, Belal A, Mehany AB, Abdelhady AA, Elhendawy MA, Radwan MM, ElSohly MA, Mahdy HA (2021). Design, molecular docking, in vitro, and in vivo studies of new quinazolin-4 (3H)-ones as VEGFR-2 inhibitors with potential activity against hepatocellular carcinoma. Bioorg Chem.

[29] Zhanzhaxina A, Suleimen Y, Metwaly AM, Eissa IH, Elkaeed EB, Suleimen R, Ishmuratova M, Akatan K, Luyten W (2021). In vitro and in silico cytotoxic and antibacterial activities of a diterpene from cousinia alata schrenk. J Chem.

[30] Imieje VO, Zaki AA, Metwaly AM, Eissa IH, Elkaeed EB, Ali Z, Khan IA, Falodun A (2021). Antileishmanial derivatives of humulene from Asteriscus hierochunticus with in silico tubulin inhibition potential. Rec Nat Prod.

[31] Mostafa A, Mahmoud SH, Shehata M, Müller C, Kandeil A, El-Shesheny R, Nooh HZ, Kayali G, Ali MA, Pleschka S (2020). PA from a recent H9N2 (G1-Like) Avian Influenza a virus (AIV) strain carrying lysine 367 confers altered replication efficiency and pathogenicity to contemporaneous H5N1 in mammalian systems. Viruses.

[32] Petersen H, Mostafa A, Tantawy MA, Iqbal AA, Hoffmann D, Tallam A, Selvakumar B, Pessler F, Beer M, Rautenschlein S (2018). NS segment of a 1918 Influenza a virus-descendent enhances replication of H1N1pdm09 and virus-induced cellular immune response in mammalian and avian systems. Front Microbiol.

[33] Reed LJ, Muench H (1938). A simple method of estimating fifty per cent endpoints. Am J Epidemiol.

[34] Gaush CR, Smith TF (1968). Replication and plaque assay of influenza virus in an established line of canine kidney cells. Appl Microbiol.

[35] Mostafa A, Kandeil A, Kutkat O, Moatasim Y, Rashad AA, Shehata M, Gomaa MR, Mahrous N, Mahmoud SH, AMM Elshaier, Y (2020). FDA-approved drugs with potent in vitro antiviral activity against severe acute respiratory syndrome coronavirus 2. Pharmaceuticals.

[36] Skariyachan S, Gopal D, Muddebihalkar AG, Uttarkar A, Niranjan V (2021). Structural insights on the interaction potential of natural leads against major protein targets of SARS-CoV-2: molecular modelling, docking and dynamic simulation studies. Comput Biol Med.

[37] Zhou J, Chan L, Zhou S (2012). Trigonelline: a plant alkaloid with therapeutic potential for diabetes and central nervous system disease. Curr Med Chem.

[38] Anwar S, Bhandari U, Panda BP, Dubey K, Khan W, Ahmad S (2018). Trigonelline inhibits intestinal microbial metabolism of choline and its associated cardiovascular risk. J Pharm Biomed Anal.

[39] Özçelik B, Kartal M, Orhan I (2011). Cytotoxicity, antiviral and antimicrobial activities of alkaloids, flavonoids, and phenolic acids. Pharm Biol.

[40] Reid SM, Westbury C, Guzys AT, Reddihough DS (2020). Anticholinergic medications for reducing drooling in children with developmental disability. Dev Med Child Neurol.

[41] Vlietinck A, De Bruyne T, Apers S, Pieters L (1998). Plant-derived leading compounds for chemotherapy of human immunodeficiency virus (HIV) infection. Planta Med.

[42] Wyde PR, Gilbert BE, Ambrose MW (1989). Comparison of the anti-respiratory syncytial virus activity and toxicity of papaverine hydrochloride and pyrazofurin in vitro and in vivo. Antiviral Res.

[43] Aggarwal M, Leser GP, Lamb RA (2020). Repurposing papaverine as an antiviral agent against Influenza viruses and paramyxoviruses. J Virol.

[44] Shih T-M, McDonough J (2000). Efficacy of biperiden and atropine as anticonvulsant treatment for organophosphorus nerve agent intoxication. Arch Toxicol.

[45] Malakar S, Sreelatha L, Dechtawewat T, Noisakran S, Yenchitsomanus P-t, Chu JJH, Limjindaporn T (2018). Drug repurposing of quinine as antiviral against dengue virus infection. Virus Res.

[46] Große M, Ruetalo N, Layer M, Hu D, Businger R, Rheber S, Setz C, Rauch P, Auth J, Fröba M (2021). Quinine inhibits Infection of human cell lines with SARS-CoV-2. Viruses.

[47] Seeler A, Graessle O, Ott W (1946). Effect of quinine on Influenza virus Infections in mice. J Infect Dis.

[48] Yamazaki Z, Tagaya I (1980). Antiviral effects of atropine and caffeine. J Gen Virol.

[49] Lipton RB, Diener H-C, Robbins MS, Garas SY, Patel K (2017). Caffeine in the management of patients with headache. J Headache Pain.

[50] Tej GNVC, Neogi K, Nayak PK (2019). Caffeine-enhanced anti-tumor activity of anti-PD1 monoclonal antibody. Int Immunopharmacol.

[51] Murayama M, Tsujimoto K, Uozaki M, Katsuyama Y, Yamasaki H, Utsunomiya H, Koyama AH (2008). Effect of caffeine on the multiplication of DNA and RNA viruses. Mol Med Rep.

[52] Batista MN, Carneiro BM, Braga ACS, Rahal P (2015). Caffeine inhibits hepatitis C virus replication in vitro. Arch Virol.

[53] Mohammadi S, Heidarizadeh M, Entesari M, Esmailpour A, Esmailpour M, Moradi R, Sakhaee N, Doustkhah E (2020). In silico investigation on the inhibiting role of nicotine/caffeine by blocking the S protein of SARS-CoV-2 versus ACE2 receptor. Microorganisms.

[54] Yim N-H, Kim A, Jung YP, Kim T, Ma CJ, Ma JY (2015). Fermented So-Cheong-Ryong-Tang (FCY) induces apoptosis via the activation of caspases and the regulation of MAPK signaling pathways in cancer cells. BMC Complement Altern Med.

[55] Wei W, Du H, Shao C, Zhou H, Lu Y, Yu L, Wan H, He Y (2019). Screening of antiviral components of Ma Huang Tang and investigation on the ephedra alkaloids efficacy on influenza virus type A. Front Pharmacol.

[56] Ebada ME (2017). Drug repurposing may generate novel approaches to treating depression. J Pharm Pharmacol.

[57] Jain N, Verma A, Jain N (2020). Formulation and investigation of pilocarpine hydrochloride niosomal gels for the treatment of glaucoma: intraocular pressure measurement in white albino rabbits. Drug Delivery.

[58] Nidorf SM, Fiolet AT, Mosterd A, Eikelboom JW, Schut A, Opstal TS, The SH, Xu X-F, Ireland MA, Lenderink T (2020). Colchicine in patients with chronic coronary disease. N Engl J Med.

[59] Schlesinger N, Firestein BL, Brunetti L (2020). Colchicine in COVID-19: an old drug, new use. Curr Pharmacol Rep.

[60] Yousefi H, Mashouri L, Okpechi SC, Alahari N, Alahari SK (2021). Repurposing existing drugs for the treatment of COVID-19/SARS-CoV-2 Infection: a review describing drug mechanisms of action. Biochem Pharmacol.

[61] McNicholl IR, McNicholl JJ (2001). Neuraminidase inhibitors: zanamivir and oseltamivir. Ann Pharmacother.

[62] Mahmoud A, Mostafa A, Al-Karmalawy AA, Zidan A, Abulkhair HS, Mahmoud SH, Shehata M, Elhefnawi MM, Ali MA (2021). Telaprevir is a potential drug for repurposing against SARS-CoV-2: computational and in vitro studies. Heliyon.

[63] Alesawy MS, Abdallah AE, Taghour MS, Elkaeed EB, Eissa H, Metwaly I (2021). In silico studies of some isoflavonoids as potential candidates against COVID-19 targeting human ACE2 (hACE2) and viral main protease (Mpro). Molecules.

[64] Nandi S, Kumar M, Saxena AK (2022). Repurposing of drugs and HTS to combat SARS-CoV-2 main protease utilizing structure-based molecular docking. Lett Drug Des Disc..

[65] Hagras M, El Deeb MA, Elzahabi HS, Elkaeed EB, Mehany AB, Eissa IH (2021). Discovery of new quinolines as potent colchicine binding site inhibitors: design, synthesis, docking studies, and anti-proliferative evaluation. J Enzyme Inhib Med Chem.

[66] Eissa IH, Khalifa MM, Elkaeed EB, Hafez EE, Alsfouk AA, Metwaly AM (2021). In silico exploration of potential natural inhibitors against SARS-CoV-2 nsp10. Molecules.

[67] Eissa IH, Dahab MA, Ibrahim MK, Alsaif NA, Alanazi A, Eissa SI, Mehany AB, Beauchemin AM (2021). Design and discovery of new antiproliferative 1, 2, 4-triazin-3 (2H)-ones as tubulin polymerization inhibitors targeting colchicine binding site. Bioorg Chem.

[68] Lemon SM, Mahmoud AA (2005). The threat of pandemic influenza: are we ready?. Biosec Bioterror.

[69] Iuliano AD, Roguski KM, Chang HH, Muscatello DJ, Palekar R, Tempia S, Cohen C, Gran JM, Schanzer D, Cowling BJ (2018). Estimates of global seasonal influenza-associated respiratory mortality: a modelling study. The Lancet.

[70] Nuwarda RF, Alharbi AA, Kayser V (2021). An overview of Influenza viruses and vaccines. Vaccines (Basel).

[71] Gubareva LV, Kaiser L, Hayden FG (2000). Influenza virus neuraminidase inhibitors. Lancet.

[72] Stiver G (2003). The treatment of influenza with antiviral drugs. CMAJ.

[73] Alasiri A, Soltane R, Hegazy A, Khalil AM, Mahmoud SH, Khalil AA, Martinez-Sobrido L, Mostafa A (2023). Vaccination and antiviral treatment against Avian influenza H5Nx viruses: a harbinger of virus control or evolution. Vaccines.

[74] Zhang T, Xiao M, Wong C-K, Mok K-PC, Zhao X, Ti H, Shaw P-C (2018). Sheng Jiang San, a traditional multi-herb formulation, exerts anti-influenza effects in vitro and in vivo via neuraminidase inhibition and immune regulation. BMC Complement Altern Med.

[75] Alam M, Nandi S (2017). Current drug design strategies for fighting against swine influenza. Curr Drug Therapy.

[76] WHO. Essential steps for developing or updating a national pandemic Influenza preparedness plan. World Health Organization; 2018. Available at https://www.who.int/publications/i/item/WHO-WHE-IHM-GIP-2018.1. Accessed 15 Nov 2023.

[77] Agban Y, Lian J, Prabakar S, Seyfoddin A, Rupenthal ID (2016). Nanoparticle cross-linked collagen shields for sustained delivery of pilocarpine hydrochloride. Int J Pharm.

[78] Iacobuzio-Donahue CA, Lee EL, Abraham SC, Yardley JH, Wu T-T (2001). Colchicine toxicity: distinct morphologic findings in gastrointestinal biopsies. Am J Surg Pathol.

[79] Deysson G (1968). Antimitotic substances. Int Rev Cytol.

[80] McAuley JL, Gilbertson BP, Trifkovic S, Brown LE, McKimm-Breschkin JL (2019). Influenza virus neuraminidase structure and functions. Front Microbiol.

[81] Cross TA, Dong H, Sharma M, Busath DD, Zhou HX (2012). M2 protein from influenza A: from multiple structures to biophysical and functional insights. Curr Opin Virol.

